# A Cold Stress‐Activated Endocrine Sentinel Chemical Hormone Promotes Insect Survival via Mitochondrial Adaptations Through the Adipokinetic Hormone Receptor

**DOI:** 10.1002/advs.202509822

**Published:** 2025-12-22

**Authors:** Jiao Zhou, Junxian Chen, Hongxia Zhang, Lu Guo, Kevin Mai, Elijah Abraham, Dilip V. Prajapati, Yue Chang, Jing Ning, Xinchen Wang, Defeng Li, Dan‐Dan Zhang, Jianghua Sun, Rebecca A. Butcher, Lilin Zhao

**Affiliations:** ^1^ State Key Laboratory of Integrated Management of Pest Insects and Rodents Institute of Zoology Chinese Academy of Sciences Beijing China; ^2^ CAS Center for Excellence in Biotic Interactions University of Chinese Academy of Sciences Beijing China; ^3^ State Key Laboratory of Microbial Diversity and Innovative Utilization Institute of Microbiology Chinese Academy of Sciences Beijing China; ^4^ Department of Chemistry University of Florida Gainesville Florida USA; ^5^ Department of Biology Lund University Lund Sweden; ^6^ College of Life Science Institute of Life Science and Green Development Hebei University Hebei China; ^7^ Shanxi Fen River Plain Farmland Shelterbelt Ecosystem National Observation Research Station Jinzhong China

**Keywords:** adipokinetic hormone receptor, ascarosides hormone, mitochondrial biogenesis, mitochondrial uncoupling, survival, diapause

## Abstract

Seasonal temperature fluctuations present a major survival challenge for insects, requiring physiological adaptation that confers resilience to cold stress. However, the hormone mechanisms governing mitochondrial adaptation to overcome cold stress remain poorly understood. Here, we identify that the endogenous ascaroside C9 (asc‐C9) acts as a chemical signal that markedly improves survival following acute cold exposure by coupling lipolysis to mitochondrial adaptation. We show that diapause larvae maintain compact mitochondrial cristae and display 2.7‐fold higher post‐chill survival compare to non‐diapause larvae, whose mitochondria undergo swelling and fragmentation. Cold stress triggers diapause‐specific accumulation of asc‐C9 (ascr#10) in the subcutaneous fat body. Exogenous asc‐C9 recruits the adipokinetic hormone receptor (AKHR) to activate the peroxisome proliferator‐activated receptor gamma coactivator 1‐alpha (PGC1α)–uncoupling protein 4 (UCP4) axis, thereby stimulating mitochondrial biogenesis, enhance uncoupled respiration, lowering the adenosine triphosphate/adenosine diphosphate (ATP/ADP) ratio, and synchronously accelerate lipid mobilization and increasing cold resilience. This asc‐C9–AKHR–mitochondria module is functionally conserved in *Drosophila melanogaster* and *Caenorhabditis elegans*, indicating that ascaroside‐mediated metabolic reprogramming is an ancient stress‐adaptation strategy. Our findings reveal a sentinel hormone‐driven lipid‐to‐mitochondria circuit that enables insects to survive extreme cold through targeted mitochondrial flexibility.

## Graphical abstract

Seasonal cold adaptation is vital for insect survival, yet the molecular mechanisms linking diapause to mitochondrial resilience remain largely unresolved. We identify ascaroside C9 (asc‐C9) as a key endocrine signal that enhances diapause survival during cold stress by activating the AKHR–PGC1α–UCP4 axis, thereby driving cold‐induced lipolysis and mitochondrial remodeling through increased mitochondrial abundance and elevated uncoupled respiration.

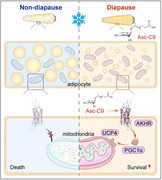

## 1 Introduction

Physiological adaptation to cold exposure is critical for insects inhabiting environments with seasonal temperature fluctuations. To survive prolonged periods of extreme winter cold, many organisms suspend feeding and enter developmental arrest (diapause), a state characterized by reduced metabolic rates and accumulated lipid reserves [1, 2, 3]. These lipid stores are primarily catabolized during transitional periods (early spring and late autumn), when insects remain non‐feeding but experience transient increases in metabolism driven by warm spells [4, 5]. Lipid metabolism thus plays a pivotal role in meeting the energy demands of insects under cold conditions. Mitochondria are central to cold stress responses: as hubs for energy homeostasis, antioxidant defense, and cell death regulation [6, 7, 8], they orchestrate key physiological adjustments. However, the upstream hormonal signals [9] governing metabolic plasticity and mitochondrial adaptations during diapause [10], and thereby determining survival following cold shock, remain poorly understood.

Ascarosides are well characterized as pheromones that modulate development, stress resistance, and behavior across diverse nematode species [11]. First identified in the free‐living nematode *Caenorhabditis elegans*, these molecules induce formation of dauer larvae, a non‐feeding developmental stage sustained by internal fat stores [12]. Subsequently, ascaroside production has been documented in various parasitic nematodes [13]. The plant‐parasitic pinewood nematode secretes ascarosides (specifically asc‐C5 with a five‐carbon side chain) to promote the pupation of its vector beetle, *Monochamus alternatus*, which carries the nematode between pine trees [14]. Furthermore, we previously showed that, in addition to nematodes, ascarosides are also produced by *M. alternatus* beetles. Beetle larvae accumulate ascarosides (specifically, asc‐C9 with a nine‐carbon side chain) in winter [14, 15]. These observations suggest conserved roles for ascarosides in nematodes and beetles, including a potential function in enhancing cold stress tolerance in *M. alternatus*.

In this study, we show that cold exposure triggers asc‐C9 production in diapause larvae, where it acts as an endocrine signaling hormone to significantly improve survival compared to non‐diapause larvae. Asc‐C9 is primarily synthesized in the subcutaneous fat body and functions as a key signal to enhance cold‐induced lipid mobilization. Mechanistically, asc‐C9 activates peroxisome proliferator‐activated receptor gamma coactivator 1‐alpha (PGC1α)–uncoupling protein 4 (UCP4)‐mediated mitochondrial biogenesis and uncoupled respiration. This pathway preserves the integrity of the mitochondrial inner membrane, thereby promoting lipolysis and enhancing survival post‐cold stress. Notably, asc‐C9 activity is dependent on the adipokinetic hormone receptor (AKHR), a receptor that mediates energy substrate mobilization in numerous insect species, indicating a mechanism conserved across insects.

## 2 Results

### 2.1 Cold‐induced Lipid Mobilization and Mitochondrial Uncoupled Respiration Promote Survival in Diapause Larvae

**FIGURE 1:**
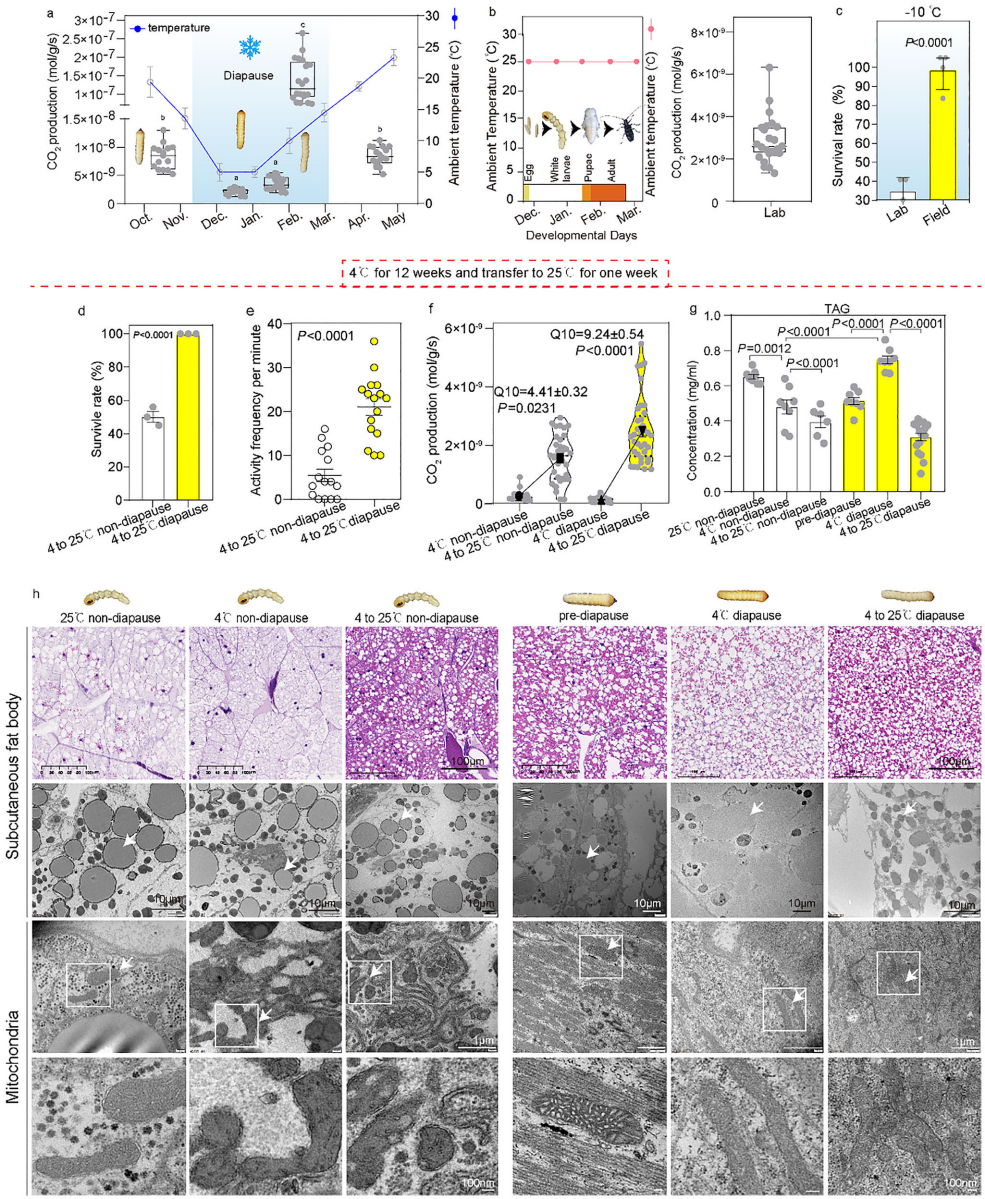
Cold‐induced lipid mobilization promotes survival in diapause larvae. (a) The whole‐body respiration rates of field‐collected larvae at environmental temperatures. The external temperature from October to May of 2018–2022 in Nanjing city was obtained from agriculture and agrometeorological data system (XMY1.4.2b) (*n* = 20). (b) The lifespan and whole‐body respiration rates of lab larvae (fifth‐stage larvae sustained at 25°C in the laboratory for 25 generations). (c) Survival rates of field and lab larvae following exposure to −10°C for 18 h and subsequent transfer to 25°C for 24 h (*n* = 4 with 20 beetles/group). d,e) Survival rates and vitality of field pre‐diapause larvae and lab population after exposure to 4°C for 12 weeks, followed by transition to 25°C for 1 week (*n* = 3 with 10 beetles/group). f) The thermal sensitive respiration Q10 in non‐diapause and diapause larvae at 4°C or transition from 4°C to 25°C (*n* = 20). (g,h) The triacyl glyceride (TAG) levels (*n* = 10–20), lipid droplets in hematoxylin and eosin‐stained sections, transmission electron microscopy of lipid droplets and mitochondria of the subcutaneous fat body in non‐diapause and diapause larvae under the following conditions: maintained at 25°C, exposed to 4°C for 12 weeks, and returned to 25°C for 1 week following cold acclimation at 4°C (*n* = 3). Data are presented as mean ± standard deviation (SD). *p* values were assessed using Student's *t* test (Figure 1a) or two‐way analysis of variance (ANOVA) (b–g).

To evaluate the effect of overwintering developmental arrest on cold stress tolerance, we compared the metabolic rate and survival of fifth‐instar *M. alternatus* larvae from two populations: a field population that had undergone natural overwintering, and laboratory population reared at constant room temperature for 35 generations (no cold exposure). From middle November to early March, field fifth‐instar larvae are in diapause (duration:120 d), with metabolic rate reaching its minimum in December and early January (at 4°C) (Figure 1a). From February to early March, as temperature rose, their metabolic rate spontaneously increased to levels exceeding the pre‐diapause stage (Figure 1a). In contrast, laboratory fifth‐instar larvae maintained stable metabolic rate at 25°C without cold stress (duration: 10–15 d) (Figure 1b). Field larvae exhibited robust cold shock resistance: 93.75% survived after 18 h exposure to −10°C (LTemp_50_ determination Figure S1a), compared to only 34.32% survived in laboratory larvae (Figure 1c). Following 24 h recovery at 25°C post‐cold shock, field larvae also showed higher activity than laboratory larvae, as quantified by movement frequency (Figure S1b).

To confirm that developmental arrest improves survival in seasonally cold environments, we simulated prolonged overwintering cold stress in the laboratory. Field pre‐diapause larvae and laboratory larvae were maintained at 4°C for 12 weeks (designated as “diapause larvae” and “non‐diapause larvae”, respectively), then transferred to 25°C for 1 week to mimic late diapause (a dormant non‐feeding state associated with transient warming). Post cold stress, 100% of diapause larvae survived and displayed greater vitality and movement than non‐diapause larvae, of which only 53.7% survived (Figure 1d,e). At 4°C, the whole‐body respiration rate of diapause larvae was significantly lower than that of non‐diapause larvae; however, after 1 week at 25°C, their respiration rate surged to levels exceeding non‐diapause larvae (Figure 1f). The temperature coefficient (Q10, a metric of metabolic rate change per 10°C temperature increase) was also higher in diapause larvae (Figure 1f). These data demonstrate that diapause‐associated naturally switch between high active metabolic rates and severe metabolic depression, which correlates with enhances survival following overwintering cold stress relative to non‐diapause larvae.

**FIGURE 2:**
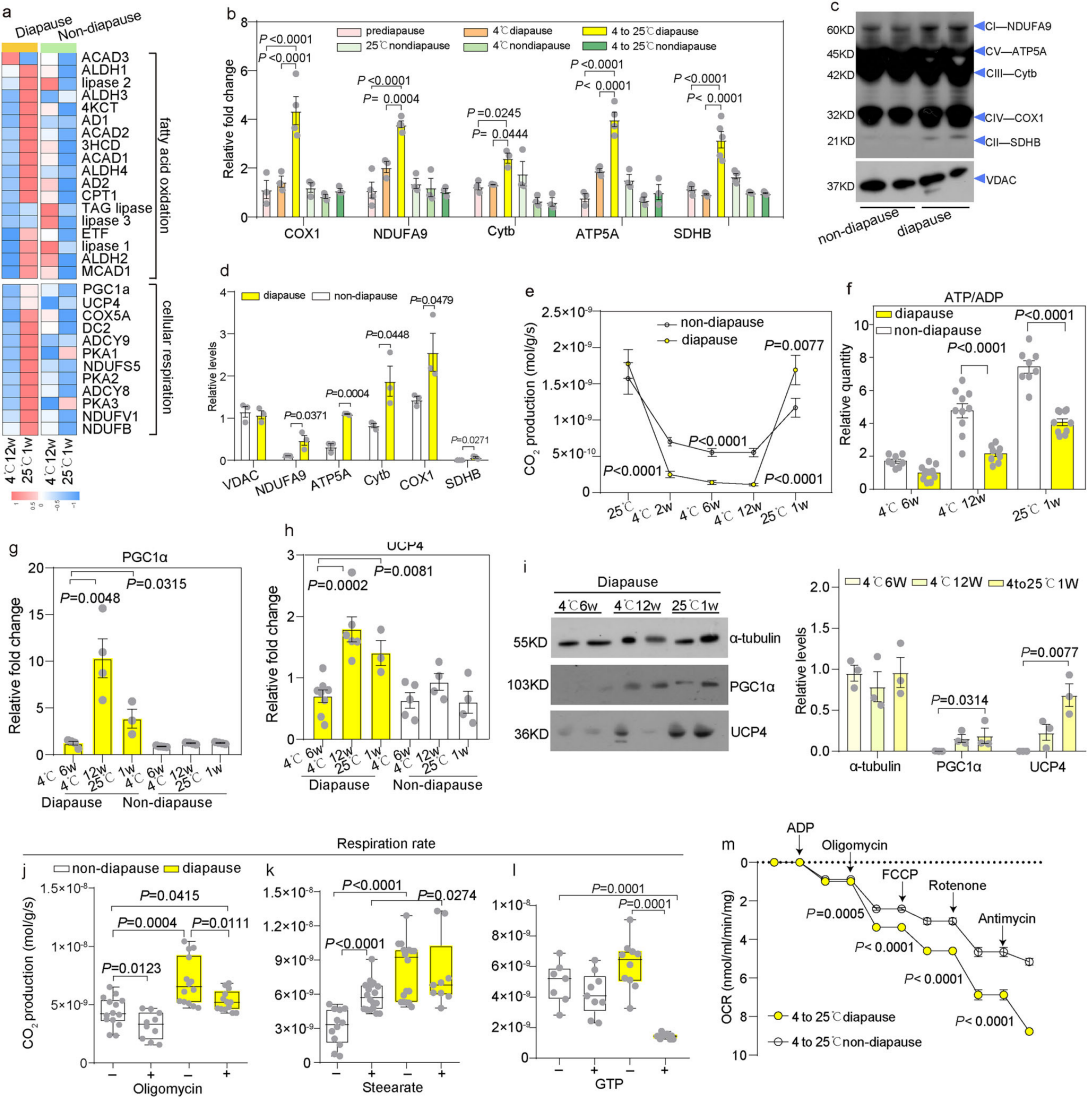
Cold induces mitochondrial uncoupling in diapause larvae. (a) Heatmap of transcripts encoding for fatty acid oxidation and cellular respiration in diapause and non‐diapause larvae at 4°C for 12 weeks, and one week at 25°C after 4°C exposure (*n* = 3). (b) Expression levels of mitochondrial DNA (mtDNA)‐encoded respiratory chain subunit genes representing Complex I–V (CI–V), including NADH dehydrogenase 1 subunit 9 (NDUFA9, CI), succinate dehydrogenase (SDHB, CII), cytochrome b (Cytb, CIII), cytochrome c oxidase subunit 1 (COX1, CIV), and ATP synthase subunit α (ATP5A, CV), in non‐diapause and diapause larvae under the following conditions: maintained at 25°C, exposed to 4°C for 12 weeks, and returned to 25°C for 1 week following cold acclimation at 4°C (*n* = 4). (c,d) Representative immunoblots of mitochondrial respiratory chain subunit proteins isolated from mitochondria of non‐diapause and diapause larvae after exposure to 4°C for 12 weeks, followed by transition to 25°C for 1 week (*n* = 4). (e) Whole‐body respiration rate (CO_2_) of non‐diapause and diapause‐larvae at 25°C, cold stress of 4°C (2, 6, 12 week), and 1 week at 25°C after 4°C exposure (*n* = 20). (f–h) Relative ATP/ADP ratio, PGC1α and UCP4 gene expression in diapause and non‐diapause larvae at 4°C for 6 weeks, 4°C for 12 weeks, and 1 week at 25°C following 4°C exposure (*n* = 5–10). (i) Protein concentrations of PGC1α and UCP4 in diapause larvae at 4°C for 6 weeks, 4°C for 12 weeks, and 1 week at 25°C following 4°C exposure (*n* = 3). (j–l) Whole‐body respiration rates after 24 h of feeding with oligomycin, stearate, or the UCP inhibitor guanosine nucleotide (GTP) in non‐diapause and diapause larvae after transition from 4°C to 25°C (*n* = 20). (m) Mitochondrial oxygen consumption rate (OCR) in non‐diapause and diapause larvae after transitioning from 4°C to 25°C. Data are presented as mean ± SD. *p* values were assessed using Student's *t* test.

We further investigated the flexible metabolic model during diapause. After 12 weeks at 4°C, diapause larvae accumulated significantly more triacylglycerol (TAG) and developed larger lipid droplets than non‐diapause larvae (Figure 1g, h; Figure S1f). Additionally, diapause larvae had a distinct free fatty acid (FFA) profile, with higher levels of C16 and unsaturated fatty acids (Figure S1c–e). Upon transferred to 25°C, diapause larvae exhibited reduced lipid droplet size, decreased TAG content (Figure 1g, h), elevated FFA concentrations (Figure S1c), and upregulated expression of transcripts involved in fatty acid oxidation and the mitochondrial electron transport system (ETS), compared to non‐diapause larvae (Figure 2a, Table S2). These observations suggest that a greater ability to mobilize lipids is essential for surviving cold stress.

Mitochondrial abundance increased significantly in larvae following the transition from 4°C to 25°C (Figure 1h; Figure S1g). Relative to non‐diapause larvae, diapause larvae showed upregulation of mtDNA‐encoded genes for ETS complexes I (NADH dehydrogenase), II (succinate dehydrogenase), III (cytochrome b), IV (cytochrome oxidase subunit 1), and V (ATP synthase) (Figure 2b). Immunoblot analysis further confirmed that diapause larvae had markedly higher levels of mtDNA‐encoded respiratory chain subunits than non‐diapause larvae (Figure 2c,d). These dynamic changes in mitochondrial abundance likely regulate the metabolic switch from lipid storage to mobilization in diapause larvae during seasonal temperature fluctuations. Notably, diapause larvae maintained intact mitochondrial ultrastructure after cold stress at 4°C (Figure 1h). In contrast, non‐diapause larvae exhibited swollen, rounded mitochondria with loss of electron density and disrupted inner mitochondrial membranes (IMM) (Figure 1h). This phenotype aligns with cold stress‐induced injury reported in insects [16], plants [17], and mammals [18, 19], suggesting that mitochondrial swelling and compromised IMM integrity are linked to cold stress mortality in non‐diapause larvae. These results indicate that mitochondrial abundance and membrane integrity during overwintering is crucial for post‐cold stress survival.

To further characterize the metabolic strategies of diapause larvae, we analyzed whole‐body respiration rates across diapause maintenance (4°C for 2, 6, and 12 weeks) and late diapause (25°C for 1 week) stages (Figure 2e). Diapause larvae had a significantly lower adenosine triphosphate (ATP)/adenosine diphosphate (ADP) ratio than non‐diapause larvae across all tested stages (Figure 2f), indicating that elevated respiration in diapause larvae is not driven by increased ETS protein abundance or ATP synthesis, but rather by an alternative mechanism such as mitochondrial uncoupling. Consistent with this, diapause larvae showed upregulated transcript and protein levels of PGC1α and UCP4 in diapause larvae across diapause maintenance and late diapause (Figure 2g–i).

To validate that elevated respiration in diapause larvae is mediated by uncoupled respiration, we treated larvae with mitochondrial complex inhibitors prior to the 4°C to 25°C transition. Oligomycin (an F_0_F_1_ ATP synthase inhibitor) suppressed respiration in both groups, but diapause larvae still maintained higher metabolic rates than non‐diapause larvae (Figure 2j). Stearic acid, an activator of the tricarboxylic acid cycle and oxidative phosphorylation [20], increased respiration in non‐diapause larvae but had no effect on diapause larvae (Figure 2k). Conversely, guanosine triphosphate (GTP), an inhibitor of mitochondrial UCPs [21], reduced respiration in diapause larvae but not in non‐diapause larvae (Figure 2l). In vitro analysis of purified mitochondria confirmed these findings: diapause larvae exhibited higher oxygen consumption rates (OCR) than non‐diapause larvae (Figure 2m). Moreover, diapause larvae displayed key hallmarks of uncoupling, including increased leak respiration (evidenced by elevated OCR following treatment with oligomycin and the uncoupler carbonyl cyanide 4‐(trifluoromethoxy) phenylhydrazone [FCCP]) (Figure 2m). Collectively, these findings demonstrate that increased mitochondrial abundance and uncoupled respiration, together with cold‐induced lipolysis contribute to survival after cold stress.

### 2.2 Asc‐C9 Induces Mitochondrial Abundance and Uncoupled Respiration in the Subcutaneous Fat Body to Enhance Cold Stress Survival

**FIGURE 3:**
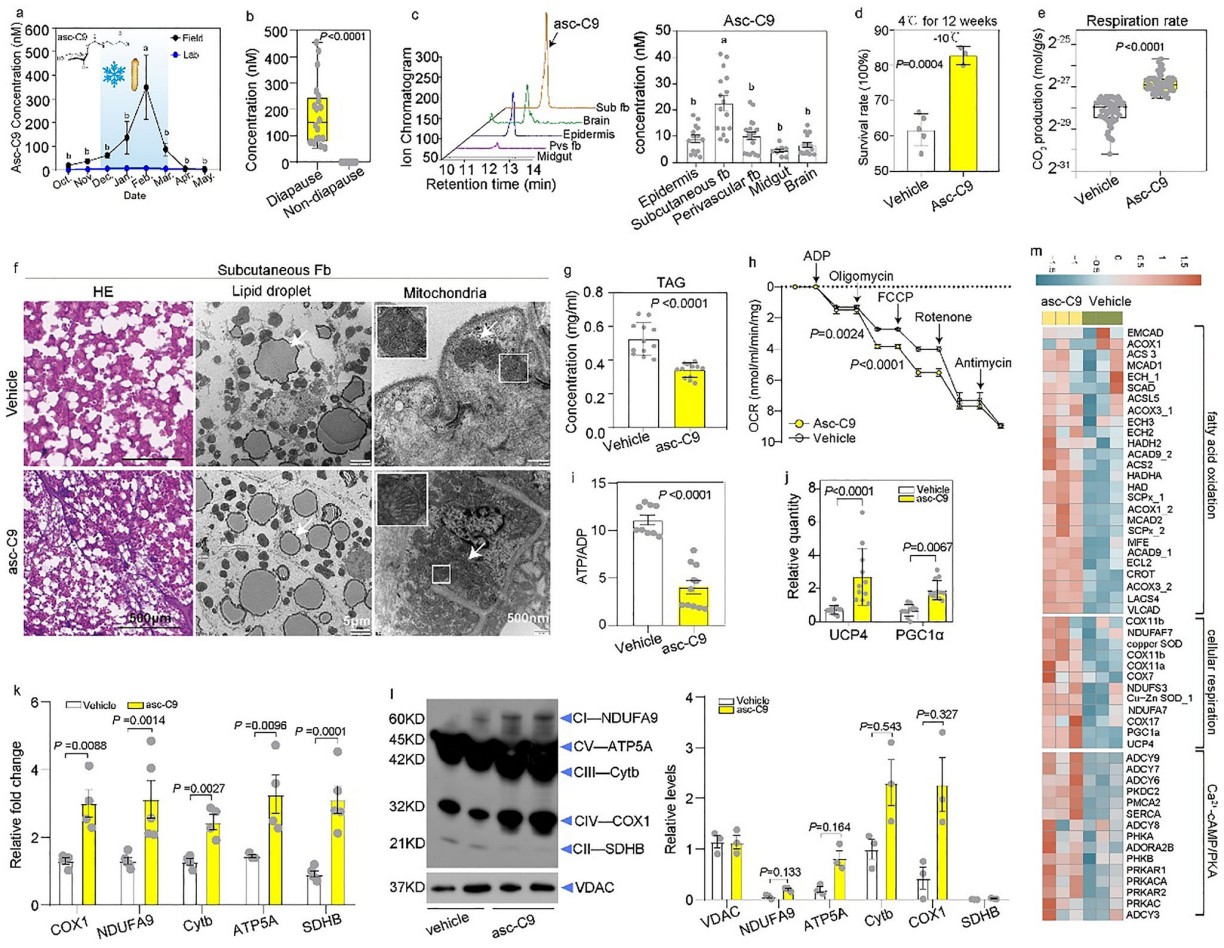
Asc‐C9‐induced mitochondrial abundance and uncoupled respiration of subcutaneous fat improves lipid mobilization. (a) Changes in asc‐C9 concentration in fifth‐stage larvae during overwintering (*n* = 4 with more than 20 beetles per larval type and time point). (b) Asc‐C9 concentration in diapause larvae and non‐diapause larvae exposed to 4°C for 12 weeks (*n* = 20). (c) Extracted ion chromatograms and content of asc‐C9 in different tissues of diapause larvae (*n* = 10). (d) Survival rates of recovered larvae injected with either vehicle or asc‐C9 under 4°C for 12 weeks, followed by a recovery period at 25°C for one week (*n* = 30). (e) The whole‐body respiration rate in recovered larvae injected with either vehicle or asc‐C9 at 25°C (*n* = 20). (f) Lipid droplets in hematoxylin‐eosin‐stained sections; lipid and mitochondria viewed via transmission electron microscopy of subcutaneous fat body in recovered larvae injected with either vehicle or asc‐C9 (*n* = 3). (g–j) TAG concentration, mitochondrial OCR, relative ATP/ADP ratio, and relative mRNA expression levels of UCP4 and PGC1α in recovered larvae injected with either vehicle or asc‐C9 at 25°C (*n* = 20). (k) Expression levels of mtDNA‐encoded respiratory chain subunit genes representing Complex I–V (CI–V) in recovered larvae injected with either vehicle or asc‐C9 at 25°C (*n* = 20). (l) Representative immunoblots of mitochondrial respiratory chain subunit proteins isolated from mitochondria of recovered larvae injected with either vehicle or asc‐C9 (*n* = 3). (m) Heatmap of transcript encoding for fatty acid oxidation, cellular respiration, and Ca2+‐cAMP/PKA pathway in recovered larvae injected with vehicle or asc‐C9 (*n* = 3). Data are presented as mean ± SD. *p* values were assessed using the Student's *t* test (b,d–j) or two‐way ANOVA (a,c).

Our previous work demonstrate that low temperatures trigger ascaroside C9 (asc‐C9) production in fifth‐instar overwintering *Monochamus* beetles [14, 15]. To explore whether contributes to cold stress survival, we first quantified its concentration in field and laboratory larvae. Asc‐C9 was detectable in field larvae but not absent in laboratory larvae. In field populations, asc‐C9 concentrations increased in autumn (coinciding with temperature decline), peaked from January to late February, and then decreased from late March to May as larvae transitioned from diapause to recovery (Figure 3a). Consistent with this, asc‐C9 was detected in larvae induced to diapause via 12‐week exposure to 4°C, but not in non‐diapause larvae (Figure 3b), with the highest concentrations localized to the subcutaneous fat body relative to other tissue (Figure 3c). When field‐collected diapause larvae were transferred to 25°C for 4 weeks to simulate recovery, asc‐C9 concentrations gradually declined and ultimately became undetectable (Figure S2b). To directly assess asc‐C9 function, we injected the molecule into larvae that had recovered at 25°C for 4 weeks (and thus exhibited low whole‐body respiration rate; Figure S2c) at a concentration matching the maximum asc‐C9 level in the subcutaneous fat body of overwintering diapause larvae. Post‐injection, asc‐C9 content in the subcutaneous fat body of recovered larvae increased threefold (Figure S2d).

Asc‐C9 injection significantly improved survival and activity in both recovered larvae and laboratory‐raised larvae following 12‐week cold stress at 4°C (Figure 3d, Figure S2a,e). Within 2 days of injection, recovered larvae exhibited elevated whole‐body respiration rates (Figure 3e), reduced lipid droplet size (Figure 3f), decreased TAG content (Figure 3g), and increased FFA levels (Figure S2f). Additionally, asc‐C9 injection upregulated the expression of mtDNA‐encoded respiratory chain subunit genes and proteins in recovered larvae (Figure 3k‐l), as well as transcripts involved in fatty acid oxidation and cellular respiration (Figure 3m). These observations indicate that asc‐C9 accelerates lipolysis and mitochondrial biogenesis. Furthermore, asc‐C9 injection enhanced respiration uncoupling in recovered larvae, as evidenced by increased mitochondrial OCR and reduced ATP/ADP ratio (Figure 3h,i). Notably, asc‐C9 unregulated the expression of UCP4 and PGC1α, key regulators of mitochondrial uncoupling and biogenesis, in recovered larvae (Figure 3j). In negative control experiments, no significant differences in metabolic rate, mitochondrial number, or ATP levels were observed between larvae injected with saline and those injected with asc‐C5 (a major pinewood nematodes pheromone; Figure S2h–j). Collectively, these results demonstrate that asc‐C9 enhances cold stress survival by increasing mitochondrial abundance and uncoupled respiration, thereby promoting lipid mobilization.

### 2.3 Depletion of Pgc1α and Ucp4 Blocks Asc‐C9‐Mediated Enhancement of Cold Stress Survival

**FIGURE 4:**
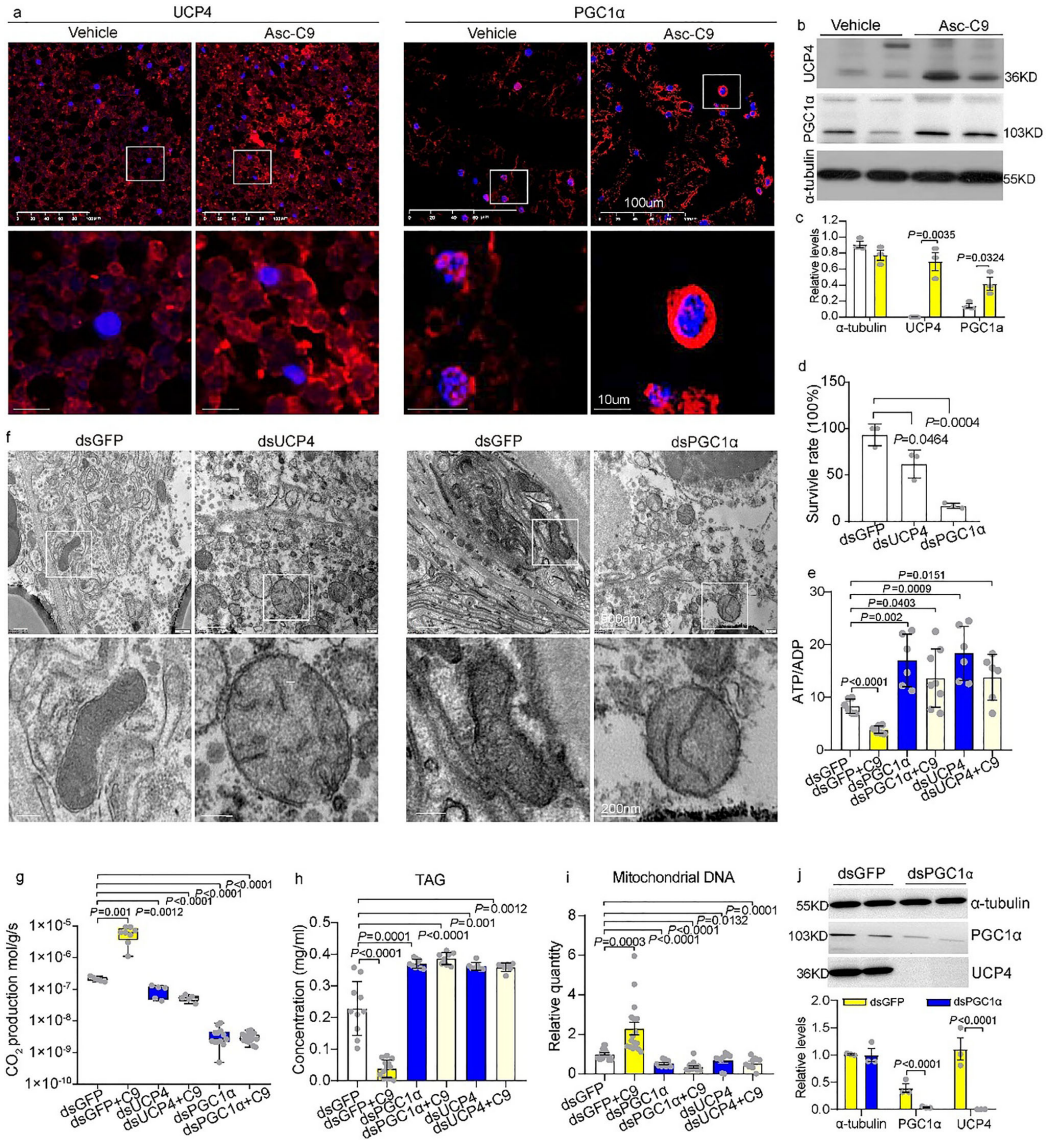
*Pgc1α* and *Ucp4* depletion blocks asc‐C9‐improved survival after cold stress. (a) Immunofluorescent staining of UCP4 and PGC1α in the subcutaneous fat body of recovered larvae injected with either vehicle or asc‐C9 (*n* = 3). (b,c) Representative western blot analysis and quantification of UCP4 and PGC1α expression in recovered larvae injected with either vehicle or asc‐C9 (*n* = 3). (d) Survival rate of GFP‐, UCP4‐, and PGC1α‐depleted diapause larvae after transition from 4°C to 25°C (*n* = 30). (e) ATP/ADP ratio of GFP‐, UCP4‐, and PGC1α‐depleted diapause larvae injected with vehicle or asc‐C9 after transition from 4°C to 25°C (*n* = 10). (f) Transmission electron microscopy showing the mitochondria of subcutaneous fat body in UCP4‐, and PGC1α‐depleted diapause larvae after transition from 4°C to 25°C (*n* = 3). (g–i) Whole‐body respiration rate, TAG concentration, and mtDNA (indicated by cytochrome c oxidase subunit 1 [COXI] as a marker for Complex IV) in GFP‐, UCP4‐, and PGC1α‐depleted diapause larvae injected with vehicle or asc‐C9 following the transition from 4°C to 25°C (*n* = 10). (j) Representative western blot analysis and quantification of UCP4 and PGC1α expression in GFP‐, and PGC1α‐depleted diapause larvae after transition from 4°C to 25°C (*n* = 3). Data are presented as mean ± SD. *p* values were assessed using Student's *t* test.

Immunofluorescence and immunohistochemistry analyses of asc‐C9‐injected recovered larvae revealed upregulated cytoplasmic UCP4 expression and nuclear PGC1α expression in the subcutaneous fat body (Figure 4a, Figure S3a,b). Western blot analysis further confirmed elevated protein levels of both UCP4 and PGC1α in these larvae (Figure 4b,c). To verify the functional necessity of PGC1α and UCP4 for asc‐C9‐dependent cold stress tolerance, we performed RNA interference to knock down *Pgc1α* and *Ucp4*. Double‐stranded RNA (dsRNA) targeting each gene was injected individually into diapause larvae (Figure S3c,d), which were then exposed to 4°C for 12 weeks followed by 1 week at 25°C. Post‐cold stress, the survival rates of *Ucp4*‐ and *Pgc1α*‐depleted diapause larvae were significantly reduced compared to larvae injected with dsGFP (Figure 4d). Mitochondria in *Ucp4*‐ and *Pgc1α*‐depleted diapause larvae exhibited swelling and loss of IMM integrity, phenotypes comparable to those in non‐diapause larvae (Figure 4f). Additionally, *Ucp4*‐depleted diapause larvae showed elevated mitochondrial reactive oxygen species (ROS) levels after cold stress (Figure S3e,f). These results indicate that PGC1α and UCP4 are critical for protecting mitochondrial structure from cold stress‐induced damage, likely via reducing mitochondrial ROS production, thereby enhancing survival. *Ucp4*‐ and *Pgc1α*‐depleted diapause larvae also displayed enlarged lipid droplets (Figure S3g,h), increased ATP/ADP level (Figure 4e), reduced whole‐body respiration rates (Figure 4g), increased TAG content (Figure 4h), and diminished mtDNA copy numbers (Figure 4i). Notably, asc‐C9 injection failed to rescue these deficits in *Ucp4*‐ or *Pgc1α*‐depleted larvae post‐cold stress (Figures 4e,g–i, and S3g,h). Moreover, *Pgc1α*‐depletion downregulated Ucp4 expression (Figure 4j), suggesting that PGC1α drives UCP4‐depended mitochondrial uncoupling. Collectively, these findings demonstrate that asc‐C9 enhances cold stress survival in diapause larvae by orchestrating metabolic and mitochondrial adaptations, primarily via the PGC1a‐UCP4 axis that regulates mitochondrial biogenesis and uncoupling.

### 2.4 AKHR Is a Target of asc‐C9

**FIGURE 5:**
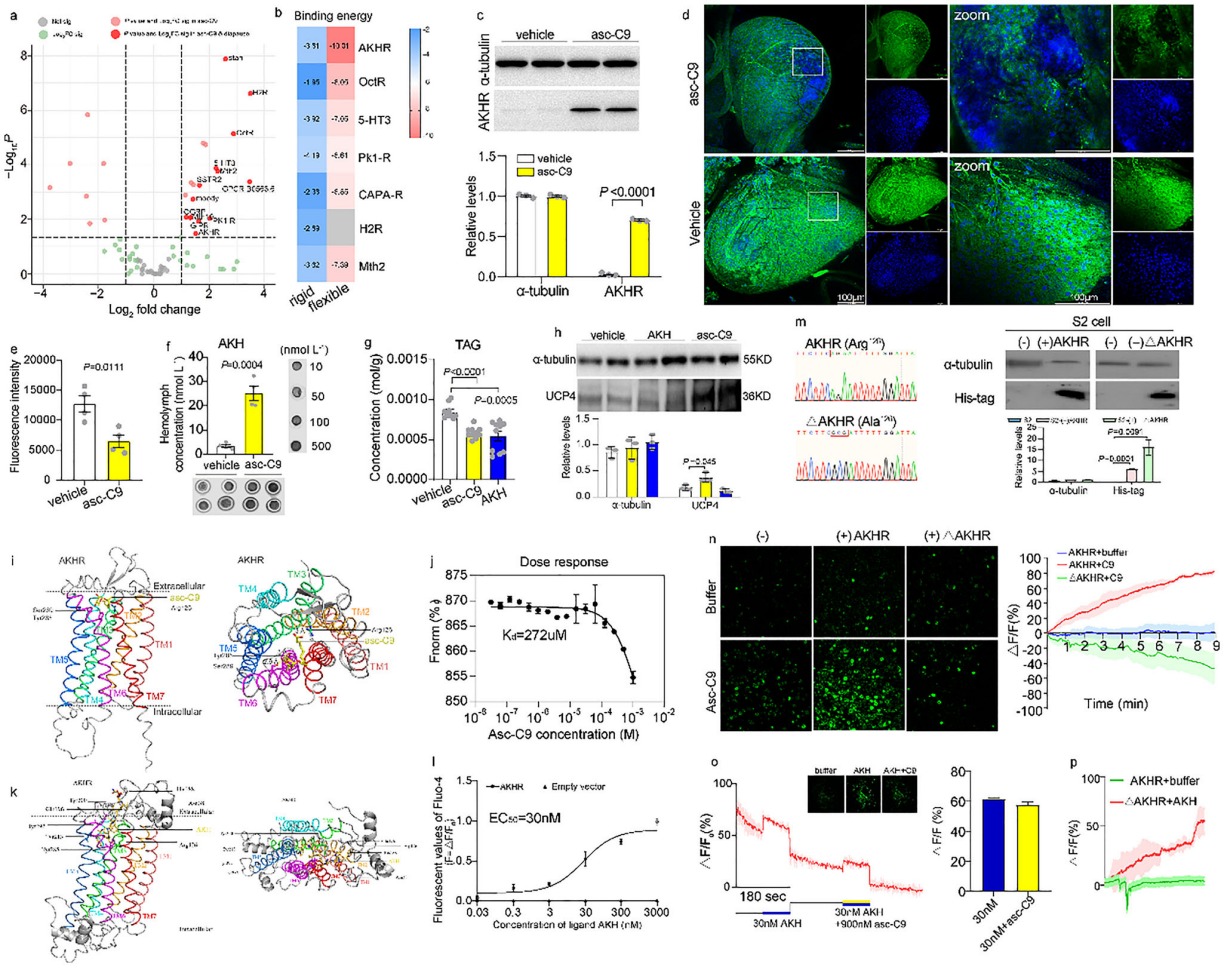
Asc‐C9‐induced uncoupled respiration via adipokinetic hormone receptor (AKHR)‐modulated lipolysis. (a) The transcript expression of GPCR genes in the fat body of recovered diapause larvae injected with either vehicle or asc‐C9 (*n* = 3). Stan functions in cell adhesion and signal reception [25, 26], H2R (histamine H2 receptor) stimulates H2‐histaminergic activation of lipolysis in WAT [27], and Mth (Methuselah) receptor modulates insulin‐like peptide secretion and extends life span [28, 29]. 5‐HT (5‐hydroxytryptamine), octopamine receptors and AKHR (adipokinetic hormone receptor) take part in cold response by regulating metabolic homeostasis [9]. 5‐HT and octopamine receptors evoke insulin‐like peptides or AKH release to regulate cold‐resistant feeding and lipolysis [30]. (b) The binding energies (kcal/mol) of the best rigid and flexible docking poses between asc‐C9 and candidate GPCRs, including AKHR, OctR, 5‐HT3, Pk1‐R, CAPA‐R, H2R, and Mth2. c) Representative western blot analysis and quantification of AKHR expression in recovered larvae injected with either vehicle or asc‐C9 (*n* = 3). (d,e) Immunofluorescence measurements of intracellular AKH levels in the corpora cardiaca of recovered larvae injected with either vehicle or asc‐C9, probed with an anti‐AKH antibody (*n* = 10). (f) Quantification of AKH hemolymph levels in recovered larvae injected with either vehicle or asc‐C9 as determined by dot blot analysis (*n* = 10). (g) TAG concentration in recovered larvae injected with vehicle, asc‐C9, and AKH (30pM) (*n* = 10). (h) Representative western blot analysis and quantification of UCP4 expression in recovered larvae injected with vehicle, asc‐C9, and AKH (30pM) (*n* = 3). (i) Lateral view and top‐down view of AKHR–asc‐C9 docking. The receptor is oriented with the extracellular side up and the intracellular side down; hydrogen‐bonding residues Arg126, Tyr285, and Ser289 are shown as sticks. Hydrogen bonds are denoted by yellow dashed lines with distances indicated. j) The binding affinity between AKHR and asc‐C9 was determined using microscale thermophoresis (*n* = 3). (k) Predicted structure of AKHR and its interaction interface with AKH peptide. Hydrogen‐bonding residues Tyr200, Tyr213, Tyr292, Tyr285, Gln196, Arg126, His188, Asn38 are shown as sticks. Hydrogen bonds are denoted by yellow dashed lines with distances indicated. l) AKH‐induced calcium flux (Fluo‐4 imaging) in AKHR‐expressing S2 cells (*n* = 3). m) Expression verification of AKHR and △AKHR in overexpressing S2 cells using anti‐His tag antibody (*n* = 3). (n) Ca^2+^ imaging and fluorescence intensity curve of S2 cells with transfected *Monochamus* AKHR and △AKHR mutant following asc‐C9 addition (*n* = 3 with three per group). (o) △F/F traces (mean ± SD) of calcium signaling sequentially responding to 30 nM AKH and AKH + asc‐C9 (900 nM). (p) Ca^2+^ imaging and fluorescence intensity curve of cells with transfected *Monochamus*△AKHR mutant following AKH addition (*n* = 3). Data are presented as mean ± SD. *p* values were assessed using Student's *t* test.

To identify potential molecular targets of asc‐C9, we analyzed G protein‐coupled receptors (GPCRs) expression in RNA‐seq datasets (Table S3), as UCP1 activity is driven by the β‐adrenergic receptors (GPCRs) –cAMP/PKA axis in mammalian brown adipocytes. Using thresholds of log_2_ fold change ≥1 and *p* < 0.05, 13 GPCRs were identified as significantly upregulated both in two comparison groups: asc‐C9–inj versus untreated recovered larvae, and diapause versus non‐diapause larvae (Figure 5a). Among these, seven GPCRs linked to metabolic homeostasis were prioritized: adipokinetic hormone receptor (AKHR), 5‐hydroxytryptamine receptor (5‐HT), octopamine receptor (OctR), pyrokinin 1 receptor (Pk1‐R), capability peptide receptor (CAPA‐R), histamine H2 receptor (H2R), and methuselah‐like receptor 2 (Mth2) (Figure 5a, Figure S4a). Of note, AKHR, 5‐HT, and OctR are known regulators of cold tolerance, feeding, and lipolysis in insects [9]. Molecular docking predictions further revealed that AKHR exhibited lower binding energy with asc‐C9 than OctR and 5‐HT (Figure 5b, Figure S4b), suggesting a higher likelihood of specific interaction with asc‐C9. Consistent with this, AKHR protein levels were significantly upregulated in recovered larvae following asc‐C9 injection (Figure 5c). Additionally, immunofluorescence staining for AKH, a neuropeptide that mediates energy substrate mobilization in many insects [22], showed a strong signal in the glandular cells of the corpora cardiaca of recovered larvae, whereas AKH staining was markedly reduced following asc‐C9 injection (Figure 5d,e). Consistently, dot‐blot analysis revealed significantly higher levels of circulating AKH in the hemolymph of asc‐C9–injected larvae (25.18 nmol L^−1^) compared with vehicle‐injected controls (3.42 nmol L^−1^) (Figure 5f). Spike recovery assays and antibody specificity validation experiments confirmed the reliability and specificity of the dot‐blot (Figure S4f–h), and the AKH titer in hemolymph was within the physiologically relevant range observed in other insects [23, 24]. These results indicate that asc‐C9 decreases AKH retention in the corpora cardiaca and enhances its release into the circulation.

To determine whether AKH administration replicates the phenotypes induced by asc‐C9, we injected AKH into recovered larvae. While AKH injection promoted lipolysis, as evidenced by reduced TAG levels, it failed to upregulate UCP4 expression (Figure 5g,h), supporting the notion of a direct interaction between asc‐C9 and AKHR. To map the precise binding interface of asc‐C9 on AKHR, we performed molecular docking simulations (Table S4). The predicted binding model of asc‐C9 to the C‐terminal intracellular domain of AKHR revealed a favorable shape complementarity between the ligand and the binding pocket (Figure 5i). Residues Arg126, Tyr285, and Ser289 of AKHR identified as key contributors to hydrogen bond formation with asc‐C9 (Figure 5i). Notably, multiple sequence alignment of AKHR orthologs across insect species confirmed that Arg126 is evolutionarily conserved (Figure S4e). Microscale thermophoresis (MST) further validated the asc‐C9‐AKHR interaction using recombinant AKHR‐GFP fusion protein and a dilution series of asc‐C9. The dissociation constant (Kd) was calculated as 272 µM, indicative of an interaction between AKHR and asc‐C9 (Figure 5j).

To validate the binding affinity and functional relevance of this interaction, we performed calcium imaging assays using an in vitro cell expression system. Transiently transfected Drosophila S2 cell lines were generated to overexpress either wild‐type AKHR or a mutant form of AKHR in which Arg126 was replaced with alanine (Arg126Ala) (Figure 5m). Cells were treated with 900 nM asc‐C9, and intracellular Ca^2+^ concentrations changes were monitored via fluorescence intensity (△F/F_0_). Asc‐C9 triggered a maximum △F/F_0_ increase exceeding 60% in AKHR‐overexpressing cells (Figure 5n), confirming AKHR activation by asc‐C9. In contrast, cells overexpressing the Arg126Ala mutant showed no significant fluorescence increase upon asc‐C9 treatment (Figure 5n), demonstrating that Arg126 is critical for asc‐C9‐medicated AKHR activation.

Whether asc‐C9 or AKH shared the same binding sites for AKHR? Molecular docking identified seven AKH binding sites (Tyr200, Tyr213, Tyr292, Tyr285, Gln196, Arg126, His188, Asn38) on AKHR (Figure 5k, Table S4). Calcium mobilization assays demonstrated a dose‐dependent activation of AKHR by AKH (EC50 = 30 nM), while no response was elicited with an empty vector (Figure 5l). It is interesting that AKH and asc‐C9 shared the same binding sites of AKHR in Arg126 and Tyr285.

To assess whether AKH and asc‐C9 compete for AKHR binding, calcium levels were measured using Fluo‐4 fluorescence in S2 cells overexpressing AKHR. Fluorescence increased following treatment with 30 nM AKH, whereas perfusion with 900 nM asc‐C9 did not reduce the signal (Figure 5o), indicating no competition between asc‐C9 and AKH for AKHR binding. In addition, the Arg126Ala mutant showed increased fluorescence in response to AKH treatment (Figure 5p). Together, these data show that asc‐C9 promotes AKHR activity and increases AKH release into circulation.

### 2.5 Asc‐C9‐Induced Uncoupled Respiration and Lipolysis Through AKHR

**FIGURE 6:**
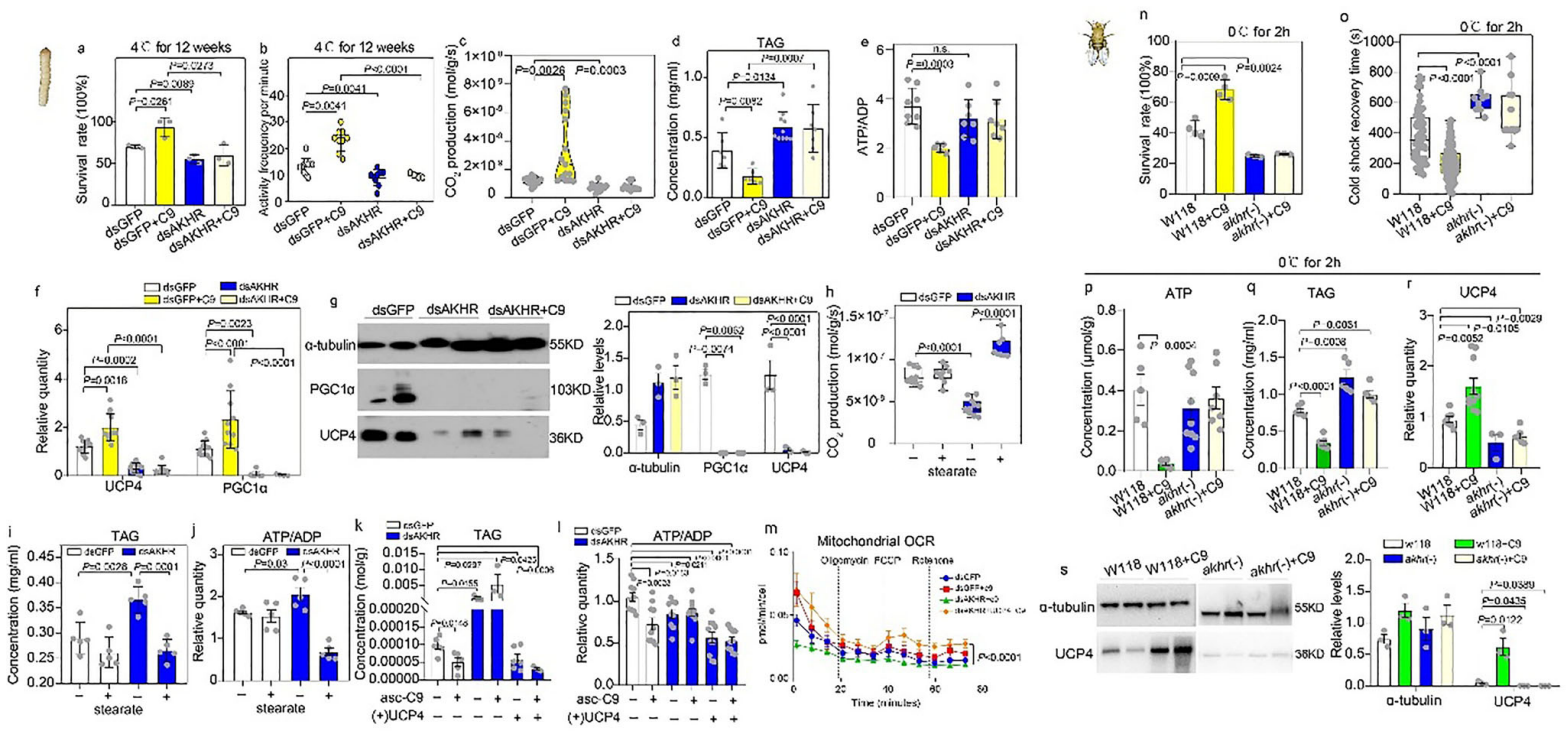
Asc‐C9‐induced lipid mobilization via AKHR signaling pathway in flies and nematodes. (a,b) Survival rate and vitality after cold shock (4°C for 12 weeks) in GFP‐, and AKHR‐depleted diapause larvae injected with vehicle or asc‐C9 (*n* = 10). (c–f) Whole‐body respiration rate, TAG concentration, ATP/ADP ratio, and UCP4a/PGC1α gene expression in GFP‐, and AKHR‐depleted diapause larvae injected with either vehicle or asc‐C9 following the transition from 4°C to 25°C (*n* = 10). (g) Representative western blot and quantification of UCP4 and PGC1α expression in GFP‐depleted, AKHR‐depleted diapause larvae, and AKHR‐depleted diapause larvae injected with asc‐C9 (*n* = 3). (h–j) Whole‐body respiration rate, TAG content, and relative ATP/ADP level of diapause larvae with GFP or AKHR knock down, injected with vehicle or uncoupling activator (stearate) (*n* = 5–10). (k–m) TAG content, relative ATP/ADP levels, and mitochondrial OCR in S2 cell treatments: dsGFP, dsGFP treated with asc‐C9, dsAKHR, dsAKHR treated with asc‐C9, dsAKHR cells overexpressing UCP4, and dsAKHR cells overexpressing UCP4 with asc‐C9 treatment (*n* = 3). (n,o) Survival rate and recovery time after cold shock at 0°C for 2 h in wild‐type and *akhr‐* mutant *Drosophila melanogaster* fed with either vehicle or asc‐C9 (*n* = 20). (p–r) ATP level, TAG content, and UCP4a expression in wild‐type, *akhr‐*mutant *D. melanogaster* fed with either vehicle or asc‐C9 under cold shock at 0°C for 2 h (*n* = 8–10). (s) Representative western blot and quantification of UCP4 expression in wild‐type, *akhr‐*mutant *D. melanogaster* fed with either vehicle or asc‐C9 under cold shock at 0°C for 2 h (*n* = 3). Data are presented as mean ± SD. *p* values were assessed using Student's *t* test.

To validate AKHR function in asc‐C9 signaling, we performed RNAi‐mediated AKHR knockdown in diapause larvae (Figure S4c), followed by 1 week of treatment at 25°C. AKHR depletion significantly reduced survival and vitality of diapause larvae post‐cold stress (Figure 6a,b), accompanied by decreased whole‐body respiration rates and FFA levels (Figure 6c, Figure S4d), and increased the TAG content (Figure 6d). Relative to dsGFP + asc‐C9 controls, dsAKHR + asc‐C9 treatment resulted in an elevated ATP/ADP ratio, confirming that asc‐C9 acts through the AKHR‐UCP4 pathway (Figure 6e). Moreover, AKHR depletion downregulated the expression of PGC1α and UCP4 both at the transcript and protein levels, and this phenotype could not be rescued by asc‐C9 injection (Figure 6f,g). Collectively, these results demonstrate that asc‐C9 induces cold‐tolerance‐associated lipolysis and uncoupled respiration via AKHR.

To investigate whether UCP4 function is dependent on AKHR signaling, we injected stearate (a mitochondrial uncoupling activator) into AKHR‐knockdown diapause larvae. This treatment failed to rescue deficits in oxygen consumption, ATP content, and TAG metabolism (Figure 6h–j), suggesting UCP4 activity requires intact AKHR signaling. We further probed the interplay between AKHR and UCP4 using Drosophila S2 cells: RNAi‐mediated AKHR knockdown significantly increased TAG content, elevated the ATP/ADP ratio, and reduced mitochondrial OCR (Figure 6k–m). Strikingly, UCP4 overexpression reversed these phenotypes, normalizing TAG accumulation, restoring the ATP/ADP ratio, and increasing mitochondrial OCR (Figure 6k–m), indicating UCP4 can counteract the metabolic consequences of AKHR depletion. These results demonstrate that AKHR is a direct target of asc‐C9, and AKHR signaling is essential for asc‐C9‐induced lipid mobilization in diapause larvae.

### 2.6 Conserved Lipid Regulation by Asc‐C9 via AKHR

To investigate whether the functional role of asc‐C9 is conserved across insect species and mediated through the AKHR, we analyzed *Drosophila melanogaster* mutants lacking *akhr* (Figure S5b). Following injection of asc‐C9, wild‐type flies exhibited enhanced survival and reduced recovery time after exposure to cold shock (0°C for 2 h) (Figure 6n,o). Under cold shock conditions, asc‐C9 injected wild‐type flies also displayed lower ATP content (Figure 6p), decreased TAG content (Figure 6q), and upregulated expression of the UCP4 gene and its encoded protein (Figure 6r,s; Figure S5). Notably, these asc‐C9‐induced effects were absent in *akhr‐* mutant flies, both in the absence and presence of asc‐C9 injection (Figure 6p–s). Collectively, these results indicate that asc‐C9 activates UCP4a‐mediated mitochondrial uncoupled respiration in Drosophila, and this pathway is necessary for supporting survival under cold shock stress.

Among the characterized ascaroside receptors in *Caenorhabditis elegans*, DAF‐38 has been reported to bind a relatively broad spectrum of ascaroside ligands [31]. To explore potential structural conservation of ascaroside‐responsive receptors, we conducted a structural alignment between the AlphaFold‐predicted model of *M. alternatus* AKHR and *C. elegans* Daf‐38. This analysis revealed a striking similarity in the overall architectures of the two receptors, including conserved structural motifs containing arginine (Arg) residues (Figure S5c,d). These conserved features suggest a potentially shared role in ligand binding between AKHR and DAF‐38.

To further investigate the functional relevance of DAF‐38 in cold stress responses, we assessed the cold sensitivity of *C. elegans daf‐38* mutants. Similar to *ucp‐4* mutants—whose role in cold stress survival has been previously established [32], the *daf‐38* mutants exhibited heightened sensitivity to cold stress (Figure S5e). Additionally, under both room‐temperature conditions and cold shock, *daf‐38* mutants accumulated higher fat stores compared to wild‐type worms (Figure S5g). Unlike wild‐type *C. elegans*, *daf‐38* mutants did not show a cold shock‐induced reduction in ATP levels (Figure S5f), suggesting that these mutants are unable to initiate mitochondrial uncoupling in response to cold stress. Together, these findings demonstrate that ascarosides enhance mitochondrial adaptation to cold shock by promoting mitochondrial uncoupling; this process, in turn, induces lipolysis and improves survival in both *Drosophila melanogaster* and *Caenorhabditis elegans*, supporting a conserved role for ascaroside‐AKHR/DAF‐38 signaling in mediating cold stress tolerance across nematode and insects.

## 3 Discussion

Identifying upstream regulators of organismal survival is critical for elucidating stress adaptation strategies in animals. In this study, we demonstrated that the ascaroside hormone asc‐C9 enhances insect survival following cold stress by activating AKHR‐PGC1α‐UCP4. This signaling cascade promotes cold‐induced lipolysis and mitochondrial adaptation, characterized by increased mitochondrial abundance and enhanced uncoupled respiration. Given the paucity of known chemical signals that govern organismal survival under stress, our findings highlight asc‐C9 as a key sentinel signal that coordinates the adaptive response to cold stress.

Our work also uncovers a novel hormonal function of asc‐C9 in beetles. In nematodes, specific ascarosides are well‐documented to induce the stress‐resistant dauer larval stage or modulate various behaviors in response to stressful environments (high population density and low food availability) [11, 33, 34]. In our study, asc‐C9 accumulated preferentially in beetle diapause larvae and conferred greater cold‐stress survival compared to non‐diapause larvae. This enhanced cold survival in diapause larvae depends on a tightly regulated metabolic balance between energy preservation and utilization mediated by asc‐C9. Long‐term cold acclimation during winter enhances the thermal sensitivity of metabolic rate and promotes lipid mobilization upon warming. Specifically, asc‐C9 triggered mitochondrial‐dependent fat mobilization, a process particularly critical during late diapause when rising early‐spring temperatures coincide with lipid utilization in the absence of feeding, thereby improving survival following cold stress. These findings indicate that diapause represent an actively regulated physiological state, which can minimize energy expenditure under cold conditions while efficiently replenishing energy reserves during transient warm periods in late winter [35, 36, 37]. Collectively, these observations suggest that asc‐C9 is a potentially conserved hormone that maintains metabolic homeostasis in stress environments across both insects and nematodes.

Our findings further indicate that mitochondrial biosynthesis and uncoupled respiration enhance mitochondrial adaptation to cold stress through the PGC1‐UCP4 axis, promoting cell survival during diapause. Consistent with previous reports, high UCP expression levels correlate positively with cold hardiness and rapid growth resumption in cold‐adapted insects [10, 38, 39, 40]. However, the precise mechanisms by which mitochondrial adaptation promotes survival under cold‐stress remain completely elucidated. In the present study, increased UCP4 expression in diapause larvae sustained low ATP production levels, an effect that ensured the efficient operation of the electron transport system following 12 weeks of incubation at 4°C. Notably, UCP4 levels remained elevated even after 1 week of rewarming at 25°C, a pattern that coincided with increased mitochondrial abundance and potential metabolic recovery at late diapause. Previous studies have linked elevated mitochondrial abundance and reduced ROS levels to facilitated metabolic recovery in diapausing insects [6, 7]. In line with this, our data showed that asc‐C9‐induced uncoupled respiration reduces ROS production in diapause larvae, which may protect the integrity of the inner mitochondrial membrane. This protection is critical for survival, as cold‐induced mitochondrial swelling (caused by inner membrane barrier dysfunction) can trigger cytochrome c release, leading to apoptosis, tissue necrosis, and ultimately organismal death [41]. Together, these results demonstrate that asc‐C9‐regulated uncoupled respiration preserves mitochondrial structural integrity and functional resilience post‐cold stress. This mitochondrial adaptation enhances metabolic flexibility, maintains redox balance and metabolic recovery, in turn, supports insect survival.

Despite the established role of UCPs in slowdown oxidative phosphorylation and antioxidant defense during cold exposure [20, 42, 43, 44, 45], the regulatory mechanisms governing UCP expression in insects remain poorly understood. Our study addressed this gap by showing that PGC‐1α activates UCP4 at the diapause termination stage, contributing to the replenishment of mitochondrial pools for cellular repair. In flies and mice, PGC1α proteins are known to be induced by environmental stimuli, such as cold exposure, exercise, or starvation, which in turn trigger diverse metabolic adaptations [46, 47, 48]. Our results demonstrate PGC1α induced the UCP4‐dependent lipid mobilization. In mammals, PGC‐1α is a key upstream regulator of UCP1 expression. It integrates energy stress signals and directly enhances the transcription of the UCP1 gene by co‐activating transcription factors such as peroxisome proliferator‐activated receptor (PPARs). This process promotes lipolysis, enhances fatty acid oxidation, and induces mitochondrial biogenesis [49, 50]. Our study revealed that in insects, PGC1α regulates UCP4 expression to promote mitochondrial biogenesis and enhance lipid mobilization. These findings suggest that the PGC1α–UCP4 axis mediates lipid utilization, exhibiting partial evolutionary conservation from insects to small mammals.

Numerous chemical signals are released either via direct intercellular contact or systemically under cold stress [9], such as capability peptides (CAPA), inotocin (ITC)‐like peptides, ion transport peptide (ITP), diuretic hormones and calcitonin (CAL), tachykinin‐related peptides (TRPs). However, studies on the neuropeptidergic control of metabolic adaptations to low temperature remain limited. Traditionally, AKH is known to govern lipolysis and diacylglycerol (DAG) mobilization during diapause maintenance [51]. Our study identifies a previously unrecognized AKH‐independent lipolytic pathway conserved across beetles and flies. Asc‐C9 binds to AKHR to activate PGC1α–UCP4–mediated uncoupled respiration, lowering cellular ATP levels and thereby stimulating fatty acid oxidation and lipid mobilization. This may because reduced ATP levels promote AKH secretion by opening ATP‐sensitive K⁺ channels, elevating intracellular Ca^2^⁺, and facilitating AKH release into the hemolymph [52]. These findings align with previous reports that UCP overexpression in Drosophila enhances AKH activity by suppressing insulin secretion and augmenting its hyperglycemic effect [52, 53, 54]. However, our results indicate that asc‐C9 does not compete with AKH for binding to AKHR. The elevated expression and activity of UCP4 observed under AKH‐independent conditions may promote enhanced lipid mobilization, meeting the increased energy demands during late diapause. Further investigations are required to elucidate the potential signaling pathway linking asc‐C9 and AKHR.

Interestingly, in *C. elegans*, the AKHR ortholog DAF‐38 has been shown to respond to asc‐C3 (ascr#5), asc‐C6‐MK (ascr#2), and asc‐△C9 (ascr#3) [55]. While DAF‐38's responsiveness to asc‐C9 remains unconfirmed, our data demonstrate that DAF‐38 is critical for cold tolerance in *C. elegans*, potentially via the upregulation of lipid metabolism. Given the structural similarity between DAF‐38 and insect AKHR, our results suggest that both receptors may enhance cold adaptation through a conserved lipid regulatory mechanism, and this possibility warrants further study.

In conclusion, we demonstrate that asc‐C9 functions as a hormone in insects to activate the AKHR‐PGC1α‐UCP4 signaling cascade. This cascade induces lipid mobilization and is essential for enhancing survival following cold stress. Such endocrine chemical signals are not only critical for insect survival and post‐winter fitness but also have potential implications for therapeutic strategies targeting metabolic disorders.

## 4 Experimental Section

### 4.1 Beetle Model

Field‐collected larvae of *M. alternatus* were obtained from *Pinus massoniana* host trees in Nanjing, Jiangsu Province, between October and May from 2018 to 2022. The whole‐body respiration rate was measured at environmental temperature to determine the stages of pre‐diapause (October to November), diapause maintenance (December to January), and late diapause (February to March) [1]. The lab larvae of *M. alternatus* were reared on the same artificial diet in a climate chamber (25°C, dark). A fresh artificial diet (200 g sawdust, 15 g agar, and 550 mL distilled water) was provided weekly. To simulate overwintering cold stress under laboratory conditions, pre‐diapause larvae (collected from October to November) and fifth‐stage lab larvae were fasted for 12 weeks at 4°C in complete darkness within a climate chamber. Following the cold exposure, both the larvae were transferred to 25°C for 1 week without feeding to mimic the seasonal warming associated with late diapause.

To test the effect of asc‐C9 on the lipid mobilization of diapause after cold stress, 5 µL of asc‐C9 (900 nM) was injected into the recovered larvae. Recovered larvae were diapause larvae collected from January with the same weight (0.3–0.4 g) and reared at 25°C for 4 weeks. The injected asc‐C9 was the same as the maximum quantity in the subcutaneous fat body of the diapause larvae from January to February (0.0045 nmol). Asc‐C9 was injected into the intersegmental membrane of larval abdomen using Hamilton Glass Syringes (1700 Series microliter syringes) with a needle having an outer and inner diameter of 0.47 and 0.13 mm, respectively (RN, 7803‐05). Metabolic indicators were measured 24 h after injection (lipid droplets were observed for 1–4d after asc‐C9 injection, with the greatest difference observed at 24 h). Asc‐C9 and asc‐C5 were synthesized and tested as previously described [14], and the same amounts were injected in the recovered larvae for metabolic detection.

### 4.2 Fly Models

*Drosophila* population w1118 (Bloomington stock number 5905) was used as a wild‐type control. These fly lines were gifts from Professor Zhou Chuan's laboratory at the Institute of Zoology of the Chinese Academy of Sciences. Flies were raised in standard medium at 25°C. *Drosophila* were reared from embryos in low‐density bottles with a standard medium containing 3.16% brown sugar, 6.32% glucose, 3.22% yeast, 1.06% agar, 7.77% cornmeal, and 15% juice supplemented with 1.75% methylparaben mix (10% methylparaben in ethanol) and 0.4% propionic acid. All percentages expressed as w/v, except for the methylparaben mix and propionic acid (v/v). Flies treated with asc‐C9 were generated by transferring w1118 flies (6–7 d old) to a medium containing 1 µM asc‐C9 for 2 d. The vehicle‐treated group was administered saline.

### 4.3 Ascaroside Concentration Determination, Treatment, and Fat Body Collection

Asc‐C9 quantification in various types and treatments of beetle larvae was performed according to a previously developed method [14]. For beetles, the concentrations were determined in larvae of the same weight (0.3–0.4 g). The asc‐C9 concentration in different beetle tissues (epidermis, subcutaneous fat body, perivascular fat body, and midgut) was determined by dissecting the tissues and extracting them in 1 mL of absolute ethanol at 25°C, followed by shaking at 150 rpm for 24 h. The extracts were filtered, dried in a vacuum centrifugal concentrator (RVC2‐18CD, Sigma), and resuspended in 200 µL of 50% (v/v) methanol in water. High‐performance liquid chromatography (HPLC)‐mass spectrometry (MS) was performed using an Agilent 1290 Series HPLC system equipped with an Agilent ZORBAX SB‐C18 column (2.1 × 150 mm, 5 µm particle diameter) connected to an Agilent 6400 Series Triple Quadrupole spectrometer in negative and ion modes [56, 57, 58]. Asc‐C9 was identified using an MS screen for the precursor ion at *m/z* = 303.1. Each experiment was performed in eight replicates.

### 4.4 Metabolic Assay

*M. alternatus* larvae were individually placed in customized respiration chambers (10 mL). The test container was installed in an incubator at 4°C, 10°C, and 25°C ± 0.5°C, which is the optimum temperature and cultivation environment for the insects. Each insect was allowed to acclimatize for 10 min before testing. After testing, the insects were exposed back to the initial conditions. Metabolic rate was estimated by measuring carbon dioxide consumption using closed‐system respirometry. The resulting carbon dioxide concentration (% CO_2_) of the samples was measured using a CO_2_ analyzer (FOXBOX, Sable Systems, USA) and recorded every second using a data acquisition system (ExpeData, Sable Systems). Samples were set at a constant flow rate of 100 mL/min, using a Mass Flow Controller (MFC2) through a column containing drierite and then to the CO_2_ analyzer. Before each trial, outdoor air was used to calibrate the analyzer. The rate of CO_2_ release (RCO_2_, in mol/g/s) was calculated as follows: (slope of CO_2_ (%/s) × exact respirometry system volume (97.18 mL) × atmospheric pressure measured (kPa) × 1000)/ (100 × 298 (K) × 8.314 × 1,000,000 × insect weight (g)). To determine the metabolic rate following the injection of mitochondrial complex inhibitors, beetles were injected with the following compounds for 24 h in separate groups: 5 mM oligomycin (blocking ATP synthase), 2.5% stearate (inducing mitochondrial uncoupling), 2 mM GTP (inhibiting mitochondrial uncoupling). The compounds were dissolved in a saline solution.

Regression equations of VO_2_ on temperature were used to calculate VO_2_^1^ at 4°C (*T*_1_) and VO_2_^2^ at 25°C (*T*_2_), which were then used to calculate Q10. This was achieved by substituting the calculated values into the equation [65].

$$Q10={\left(\frac{{V_{O}}_{2^{2}}}{{V_{O}}_{2^{1}}}\right)}^{\frac{10}{\left(T2-T1\right)}}$$

### 4.5 Mitochondrial Oxygen Consumption

Fresh mitochondria from the fat bodies of beetle larvae were extracted using a mitochondrial extraction kit (EX2620‐100T, Solarbio). Two larvae were pooled per group, and the extraction was performed at 4°C using a 1 mL Dounce tissue grinder. Protein concentration was measured using a bicinchoninic acid protein assay kit (CW0014S, CWBIO). The mitochondrial OCR of beetle larvae was measured using a Hansatech Oxytherm Control Unit. Freshly isolated mitochondria were resuspended in respiration buffer (120 mM KCl, 5 mM KH_2_PO_4_, 3 mM HEPES, 1 mM EGTA, 1 mM MgCl_2_, and 0.2% BSA (w/v), pH 7.2). The following substrates for mitochondrial respiratory chain complexes were added sequentially: 10 mM pyruvate, 2 mM malate, and 10 mM proline, along with 5 mM ADP, uncoupler, and inhibitors of oxidative phosphorylation (5 µM oligomycin A, 1 µM FCCP, 2 µM rotenone, 2.5 µM antimycin A). FCCP was titrated five times, adding 1 µL per titration. The total reaction volume in the respiratory chamber was 1 mL. Oxygen consumption signals were recorded at 25°C. After calibration based on protein concentration, the mitochondrial OCR was expressed as nmol/ml/min/mg.

### 4.6 Electron Microscopy

Fresh larval fat bodies were fixed in 2.5% glutaraldehyde, washed with 1× phosphate‐buffered saline (PBS), and post‐fixed in 1% osmium tetroxide. The samples were then dehydrated in 30% and 50% ethanol, followed by immersion in uranyl acetate solution at 4°C in the dark for 12 h. Subsequently, tissues were further dehydrated using a graded ethanol series (80%, 90%, and 100%). After a 20‐min treatment with acetone, the tissues were infiltrated with a graded series of embedding medium mixtures for 1, 3, and 5 h, respectively. The samples were then embedded in molds, sectioned using an ultramicrotome, stained with lead citrate, and examined under a transmission electron microscope.

### 4.7 Mitochondrial DNA Copy Number and ATP/ ADP Ratio

Mitochondrial DNA copy number was assessed by using quantitative polymerase chain reaction with DNA as a template, using primers against mtDNA‐encoded genes for ETS complexes I (NADH dehydrogenase), II (succinate dehydrogenase), III (cytochrome b), IV (cytochrome oxidase subunit 1), and V (ATP synthase), and normalized to RpL32. The ATP/ADP ratio was measured using a highly stable ATP Assay Kit (Abcam, ab287863) following the manufacturer's instructions.

### 4.8 RNAi Experiments

dsRNA was prepared using a T7 RNAi Transcription Kit (P1700, Promega) following the manufacturer's instructions. Using a Hamilton Glass Syringes (1700 Series microliter syringes, RN, 7803‐05), 30 nM of the corresponding dsRNA was injected into the middle segment of the back of the beetle larvae, and samples were collected 3 d after injection. All primers used for dsRNA preparation are listed in Table S1–S4. To assess thermal‐sensitive metabolism, dsRNA was injected into diapause larvae collected in February, weighing approximately 0.3–0.4 g. After 3 d of dsRNA injection, either asc‐C9 or saline was injected, and the larvae were then transferred to 25°C for 1 week. S2 cell interference involves adding 15 µg of dsRNA directly into the culture medium, followed by a media change 24 h later.

### 4.9 Immunocytochemistry of AKH in Corpora Cardiacas

Corpora cardiaca tissues were dissected from beetle larvae in cold PBS and fixed in 4% paraformaldehyde (PFA) for 50 min. The tissues were then blocked with 3% normal goat serum (NGS) in PBS containing 0.3% Triton X‐100 (PBST/NGS) for 50 min at room temperature (RT). Corpora cardiaca were sequentially incubated with the primary antibody against AKH (1:20, ABD‐063, Jena Bioscience) in 1% bovine serum albumin (BSA) containing 0.3% Triton X‐100 (PBST/BSA), followed by incubation with the secondary antibody (1:50, Alexa Fluor 488, ab150077) in 1% PBST/BSA overnight at 4°C [23]. After each antibody incubation, tissues were washed four times with PBST. Nuclei were counterstained with DAPI (1 µg/mL) for 10 min, and samples were mounted in anti‐fade mounting medium (Invitrogen). Images were acquired using a Leica STELLARIS confocal microscope with LAS X software. AKH73 peptide levels were quantified using Image‐Pro Plus software.

### 4.10 Dot‐blot Assay

Hemolymph was collected from five live larval beetles and pooled as one sample; four biological replicates were prepared in total. To minimize peptide degradation, hemolymph was processed immediately on ice. Samples were centrifuged at 2,500×*g* for 5 min at 4°C. For dot‐blotting, 5 µL hemolymph per sample was spotted onto a 0.2 µm nitrocellulose membrane (GE Healthcare) and air‐dried at room temperature (RT) for 20 min. The *M. alternatus* AKH peptide (MaAKH; sequence: pQVNFSPNWGa) was custom‐synthesized (Beijing Shengyuan Kemeng Gene Biotechnology Co., Ltd.). Sequence alignment of MaAKH and the antibody AKH (ABD‐063, Jena Bioscience) using ClustalX and GenDoc revealed 75% identity (Figure S4f). Matrix‐matched standards were prepared by diluting synthetic MaAKH into hemolymph from recovered larvae (which showed low endogenous AKH in preliminary measurements). Serial dilutions (10, 50, 100, and 500 nM) were applied to the same membrane to generate a standard curve. Spike‐recovery assays were also performed by adding known amounts of synthetic MaAKH to hemolymph of recovered larvae. The dot‐blot showed ∼55% recovery of exogenous MaAKH added to hemolymph samples (Figure S4g). Membranes were then fixed in 4% paraformaldehyde (PFA) in PBS for 20 min, blocked in 5% BSA in PBST (PBS containing 0.1% Tween‐20) for 1 h at RT, and incubated overnight at 4°C with anti‐ AKH primary antibody (1:500 in 5% BSA/PBST; ABD‐063, Jena Bioscience) [23]. After four times washes in PBST, membranes were incubated with goat anti‐rabbit IRDye 800CW secondary antibody (LI‐COR, 926‐32211; 1:2500 in 5% BSA/PBST) for 1 h at RT. Signals were captured on an Odyssey F imaging system (LI‐COR), and dot intensities were quantified using Image‐Pro Plus software. To confirm the specificity of the AKH antibody, hemolymph samples collected after asc‐C9 injection were divided into two groups. For the control group, dot‐blot analysis was performed using the AKH antibody alone. For the experimental group, the AKH antibody was pre‐incubated with 1.3 µM synthetic MaAKH peptide for 30 min at 4°C before application. Pre‐incubation with MaAKH pricompetitively blocked the antibody binding sites, thereby reducing signal intensity. An 85.97% reduction in the dot‐blot signal compared with the control verified the high specificity of the AKH antibody toward the MaAKH peptide (Figure S4h).

### 4.11 Transfection of S2 Cells and Ca^2+^ Imaging

Plasmid construction was performed using the ClonExpress ultra one step cloning kit and Mut Express universal fast mutagenesis kit (Vazyme, C117‐01, C216‐01). S2 cells were grown to approximately 80%–90% confluence as observed under a light microscope. The cells were dislodged from the flask by washing with the medium. In total, 1× 10^6^ S2 cells were suspended in 3 mL serum‐free medium (SFX, Hyclone, SH30278.02) in each well of a Nunclone six‐well tissue culture plate (Corning). Confluent cells (80%–90%) were briefly transfected with 2.0 mg pMT‐V5‐HisA‐AKHR in Cellfectin II reagent (Gibco, 10362100) according to the manufacturer's instructions. The medium containing the plasmid DNA and Cellfectin II was removed after incubation for 24 h.

After the cells were transfected for 48 h, the medium was removed and the cells were washed thrice with Hank's balanced salt solution (without Ca^2+^). The cells were subsequently cultured at 37°C in the dark for 30 min in the presence of 5 µM Fluo‐4‐AM (Invitrogen, F14201) and were stimulated with 900 nM asc‐C9. Calcium imaging was performed using two‐photon microscopy. A luminescence assay was performed by measuring intracellular Ca^2+^ mobilization in the transfected S2 cells. The resulting luminescence was measured for 20 s at half‐second intervals using a Nikon A1R MP Multiphoton System (Berthold Technologies, Bad Wildbad, Germany). A concentration‐response curve was calculated by log fitting. All experiments were conducted in triplicate.

### 4.12 Structural Prediction, Homology Analysis, and Molecular Docking

The three‐dimensional structure of AKHR and candidate receptors was predicted using AlphaFold 3 [59]. Protein sequences of receptors were retrieved from BFD, UniClust30, UniRef90, and MGnify databases using HHblits or JackHMMER with default settings. Low‐confidence N‐ and C‐terminal residues (pLDDT < 70) were removed from the final models. Structural similarity was assessed using the DALI server against the PDB.

Homologous sequences of AKHR and DAF‐38 were identified with ConSurf using PSI‐BLAST (default settings) against the UniRef90 database, aligned with MAFFT [60], and analyzed for conservation using the Bayesian method.

For docking analysis between AKHR and asc‐C9, the AlphaFold‐predicted AKHR model was prepared by adding hydrogens and Kollman charges, and docking was performed with AutoDock Vina [61]. The grid box (44 × 32 × 36 Å, spacing 0.375 Å) encompassed the transmembrane hydrophobic cavity. The ligand Asc‐C9 was obtained from PubChem and prepared using AutoDock Tools. For docking analysis between AKHR and asc‐C9, AKH model prediction was constructed using Chai‐1 [62]. Alanine scanning was performed with FoldX [63]. Docking analysis was carried out using Hdock [64]. All structures and docking results were visualized in PyMOL.

### 4.13 Microscale Thermophoresis Binding Assay

The binding affinity between asc‐C9 and AKHR was determined by microscale thermophoresis (MST). The lysates of S2 cells briefly transfected with GFP‐tagged AKHR were prepared. Several concentrations of the required ligand (30.5 to 1 mM) were incubated with 30 µM cell lysate for 20 min in assay buffer (50 mM Tris‐HCl pH 7.4, 150 mM NaCl, 10 mM MgCl_2_, 0.05% Tween‐20). The sample was loaded into Nano Temper glass capillaries, and microscale thermophoresis was performed using 20% LED power and 80% MST power. Dissociation constant (Kd) was calculated using the Nano Temper software from duplicate reads of triplicate experiments. Nano Temper monolith NT.115 was used for the analysis.

### 4.14 Mitochondrial Isolation

Tissues were gently crushed in a glass tube with chilled mitochondrial isolation buffer (MIB) (210 mM of d‐Mannitol, 70 mM of sucrose, 5 mM of HEPES, 1 mM of EGTA, and 0.5% (w/v) of fatty acid‐free BSA, pH 7.2) and centrifuged at 800×*g* for 10 min at 4°C to remove nuclei and debris. The supernatant was then spun at 8000×*g*, for 10 min at 4°C. The pellet, containing the mitochondria, was washed once in fresh MIB and then the total protein content of the resulting pellet was determined by the Bradford assay.

### 4.15 Mitochondrial Oxygen Consumption Measurements

Cells were resuspended in mitochondrial assay solution (MAS) (220 mM of d‐Mannitol, 70 mM of sucrose, 10 mM KH_2_PO_4_, 5 mM MgCl_2_, 2 mM HEPES, 1 mM of EGTA, and 0.2% of fatty acid‐free BSA, pH 7.2). Oxygen consumption rate was measured using the Agilent Seahorse XFe96 Analyzer (Agilent Technologies). Oxygen consumption rate measurements of beetle fat body were performed followed by the sequential addition of 20 µL of oligomycin (2.5 µM), 20 µL of FCCP (2 µM) and 20 µL of rotenone /antimycin A (0.5 µM) (Agilent Technologies, 103015‐100).

### 4.16 Statistical Analysis

Comprehensive statistical analyses were performed using SPSS 17.0. Data were expressed as mean ± standard deviation. Two‐tailed *t*‐test was used for single comparisons and analysis of variance (ANOVA) with Tukey's post hoc tests for multiple comparisons. Data graphing was done using the software GraphPad Prism 8.0.

## Author Contributions

L.Z. conceived and supervised the study. J.Z., J.C., H.Z., K.M., E.A., and D.P. designed and performed the experiments. L.G. and D.L. conducted protein structure prediction and molecular docking analyses. W.X., J.N. and Y.C. assisted with resource acquisition and data analysis. J.Z. wrote the main manuscript. L.Z., R.B., D.Z., and J.S. reviewed and edited the manuscript.

## Conflicts of Interest

All authors declare no conflict of interest.

## Supporting information




**Supporting File 1**: advs73482‐sup‐0001‐SuppMat.docx.

**Supporting File 2**: advs73482‐sup‐0002‐Supplementary‐Table1.xlsx.

**Supporting File 3**: advs73482‐sup‐0003‐Supplementary‐Table2.xlsx.

**Supporting File 4**: advs73482‐sup‐0004‐Supplementary‐Table3.xlsx.

**Supporting File 5**: advs73482‐sup‐0005‐Supplementary‐Table4.xlsx.

## Data Availability

The data that support the findings of this study are openly available in [Figureshare] at [https://doi.org/10.6084/m9.figshare.28573898], reference number [0].
