# Supplementary material for: A Cold Stress‐Activated Endocrine Sentinel Chemical Hormone Promotes Insect Survival via Mitochondrial Adaptations Through the Adipokinetic Hormone Receptor
Source: Adv Sci (Weinh). 2025 Dec 22;13(10):e09822. doi: 10.1002/advs.202509822 (PMC12915204; doi:10.1002/advs.202509822)
Supplement: Supplementary file 1 — Supporting File 1: advs73482‐sup‐0001‐SuppMat.docx. [file ADVS-13-e09822-s001.docx]

**Supplementary Methods**

*C. elegans Models：C. elegans* variety Bristol, strain N2 (wild type), *daf-38 (ok2765)*, and CY121 *ucp-4 (ok195)* mutant worms were grown at room temperature (20–23°C) on nematode growth agar plates seeded with OP50 bacteria.

*Cell Culture:* The Drosophila Schneider 2 (S2) cells (RRID: CVCL_Z232) were a kind gift of the research group of Professor Zou Zhen, Institute of Zoology, CAS. Drosophila S2 cells were cultured in insect medium (Gibco) supplemented with 10% fetal bovine serum (FBS, Hyclone) and antibiotics (penicillin and streptomycin, Invitrogen) at 27 ℃.

*Cold Stress Survival Assay, Oil Red O Staining, and ATP Analysis in C. elegans:* To examine the survival of *C. elegans* under cold stress, 50–100 synchronized young adult-stage worms were prepared. Synchronized egg laying was performed by placing several young adult worms on a 6 cm agar plate seeded with OP50 bacteria and then removing the worms after 2 h. Once the progeny reached the young adult stage, they were transferred to a 2.5 °C incubator for 24 h and then to a 25 °C incubator for 1 h for recovery. Worms were scored alive if they responded to a gentle tap with a platinum wire pick. Oil Red O staining was performed to assess fat storage. Synchronized worms were collected at the young adult stage and washed with M9 buffer to remove bacteria. The samples were dehydrated and permeabilized with 40% 2-propanol for 3 min, followed by treatment with Oil Red O (3 mg/mL in 60% 2-propanol) and allowed to rotate in the dark at 25 °C. After 2 h, the samples were washed with M9 to remove the excess dye. For ATP determination, larvae were suspended in extraction medium (0.1 M NaOH, 0.5 mM EDTA) and incubated at 60 °C for 20 min, followed by freezing at -80 °C. The lysates were diluted and added to the assay solution (250 mM glycylglycine, pH 7.4, 2 mM EGTA, 2 mM MgCl_2_, 0.4 g/L BSA, 7.5 mM DTT) with 0.015 mM luciferin and 10 mg/mL luciferase. The reaction was initiated by adding the samples and was incubated for 10 s. The light output was measured using a BMG Labtech CLARIOstar Plus Microplate Reader^[^[^1^](#_ENREF_1)^]^. ATP standards were run in parallel to determine relative ATP levels in each sample. Bradford assay was performed to quantify the approximate amount of protein in each sample, relative to the standards run in parallel.

*Measurement of Survival Rate after Cold Stress:* In the mortality experiments, fifth-instar larvae from both field and laboratory populations were exposed to −10°C for 18 hours in incubator. In laboratory survival experiments, beetle larvae from each treatment were exposed to 4°C for 12 weeks in an incubator. After that, all larvae were placed in a climate chamber (25°C, light: dark = 12:12 h) to recover for 1d. Death was assessed by the absence of mandibular or body movement when larvae were stimulated with a needle. Twenty individuals were tested in each treatment. Each treatment had three replicates.

*Low Temperature Tolerance and Discriminating Temperature Determination:* To establish a standard cold shock exposure (discriminating temperature) for comparison of larval survival after different pretreatments, pre-diapause larvae were transferred directly from their rearing conditions (25°C, 14L:10D) to -8°C, -10°C, and -12°C for 12h, 18h or 24h. The larvae were placed in climatic incubators (Hardy Technology International Ltd., Chongqing, China). A thermometer inside was used to check whether the temperature needed to be adjusted prior to each heat-shock treatment. Beetles were returned to 25°C and survival was evaluated after 24h.

*Hematoxylin-Eosin (HE) Staining, Immunofluorescence, Immunohistochemistry and Immunoblot Analysis:* For HE staining, tissues were fixed in 4% paraformaldehyde, dehydrated through a graded ethanol series, cleared in xylene, and embedded in paraffin blocks. Sections of 5 μm thickness were cut and mounted onto glass slides. After HE staining, coverslips were applied using neutral resin.

Immunofluorescence was performed on 7 μm paraffin-embedded sections prepared using a Leica CM1900 (Germany). The sections were incubated at 37 °C for 10 minutes, fixed in cold acetone for 10 minutes, and then washed three times with PBS for 5 minutes each. Samples were blocked in 0.1% Triton X-100 and 5% bovine serum albumin (BSA) for 1 hour at room temperature, followed by incubation overnight at 4 °C with primary antibodies against UCP4 and PGC1α (custom-made polypeptide antibodies, 10 μg/mL) diluted in the same blocking buffer. α-Tubulin was used as a reference marker in this study. After primary antibody incubation, sections were washed five times with PBS (5 min each), and incubated with Alexa Fluor 594-conjugated goat anti-rabbit IgG secondary antibody (1:3000; Thermo Fisher, A-11012) for 30 minutes at room temperature. Nuclear staining was performed using DAPI (1 μg/mL) for 10 minutes. Finally, sections were mounted using anti-fade mounting medium (Invitrogen) and imaged using a Leica STELLARIS confocal microscope and LAS ⅹ software.

For immunohistochemistry, sections were blocked with 2.5% normal goat serum in PBST (PBS + 0.1% Tween-20) for 1 hour at room temperature. Primary antibodies against UCP4 and PGC1α (custom-made, 10 μg/mL) were applied overnight at 4 °C. After three PBST washes, sections were incubated with SignalStain Boost IHC secondary antibody (anti-rabbit, Cell Signaling, 8114) for 1 hour at room temperature. Signal detection was performed using a DAB Kit (Vector Laboratories) following the manufacturer’s instructions. Sections were counterstained with hematoxylin and eosin (H&E), and images were captured using the EVOS FL Color Imaging System. Lipid area and signal intensity were quantified using ImageJ2 software.

For immunoblot analysis, samples for immunoblotting analysis were lysed by RIPA (Beyotime, P3001), separated by 4-15% gradients SDS polyacrylamide gels (Bio-Rad), and electro transferred to polyvinylidene difluoride membranes (Invitrogen). The PVDF membranes were blocked with 5% skim milk and incubated with primary antibody at 4 °C overnight. After being washed three times with PBS supplemented with 0.1% Tween 20 (Solarbio, T8220), the PVDF membranes were incubated with HRP-conjugated secondary antibodies. The signals were detected by NcmECL Ultra (NCM Biotech, P10300). Primary antibodies included: UCP4, PGC1α and AKHR (for beetle, a custom-made antibody, 1μg/mL) overnight at 4 °C, α-tubulin (Cell Signalling Technology, 11H10, 1:5000), VDAC1 (Abcam, ab14734, 1:5000), His-tag (Cell Signalling Technology, 12698, 1:5000), total OXPHOS rodent WB antibody cocktail (Abcam, ab110413, 1:250), anti-rabbit IgG (H+L) HRP conjugate (Promega, W4011, 1:2500), anti-mouse IgG (H+L) HRP conjugate (Promega, W4021, 1:2500).

*Quantitative Real-Time PCR (RT-qPCR):* RT-qPCR was performed in triplicate using the SYBR premix ExTaq (Takara, Japan) and MX3000P thermal cycler (Stratagene, USA). Thermal cycling was performed at 95 °C for 30 s, followed by 40 cycles of 95 °C for 5 s, 55 °C for 30 s, and 72 °C for 30 s. All PCR reactions were performed in triplicate, and to check for specificity of the PCR reactions, melting curves were analyzed for each data point. α- tubulin of *M. alternatus* was used as the internal control for normalization. The primers used for this study are included in Table S1. The expression value was calculated using the ΔΔCt method and normalized to α-tubulin expression level. Results were shown as mean ± SEM of three independent biological repeats.

*Determination of TAG and FFA Levels: M. alternatus* fat were homogenized in 100 μL PBS containing 0.5% Tween-20 and were incubated at 70 °C for 5 min. Then, the samples were incubated with triglyceride reagent (Sigma) for 30 min at 37 °C. Following centrifugation, samples were transferred into 96-well plates, incubated with free glycerol reagent (Sigma) for 5 min at 37 °C, and then assayed using SpectraMax Plus384 with a wavelength of 540 nm.

Larval fat bodies were frozen in liquid nitrogen and ground in 1 mL of a 2% H_2_SO_4_/98% methanol solution. Samples were sealed with a cap and incubated at 80 °C for 1 h. After the addition of 0.3 mL of hexane and 1.5 mL of H_2_O, the fatty acid methyl esters were extracted into the hexane layer by shaking and then were centrifuged at 5,000 × g for 10 min. Samples of the organic phase were carried out by means of GC-MS on an Agilent Technologies 6890N GC-5973N mass selective detector. The GC was equipped with a HP-5MS column [60 mm × 0.25 mm (i.d.); film thickness 0.25 μm] (J&W Scientific). One-microliter samples were injected at a temperature of 280 °C; the GC-MS transfer line temperature was 280 °C, ion source 230 °C, and quadrupole 150 °C. All compounds were analyzed with 70 eV nominal electron energy and a scan range of 35–400 atomic mass units, with a solvent delay of 3 min. After injection, the column temperature was held at 50 °C for 0.5 min and then was increased to 200 °C at 5 °C/min, followed by an increase to 240 °C at 2 °C/min and then an increase to 250 °C at 5 °C/min and held for 10 min. Subsequently, the temperature was increased further to 280 °C at 3 °C/min and held for 3 min. Compounds were identified by comparing their retention time with those of authentic reference compounds and comparing the spectra with that of mass spectral library NIST02 (Rev. D.04.00; Agilent Technologies).

*Measurement of Mitochondrial Reactive Oxygen Species:* The mitochondrial superoxide levels were assessed using MitoSOX™ Red Mitochondrial Superoxide Indicator (M36008, Thermo Fisher Scientific) following the manufacturer’s instructions. Briefly, the MitoSOX Red stock solution was prepared by dissolving the reagent in DMSO (D12345, Thermo Fisher Scientific) and subsequently diluted to 500 nM MSR reagent in HBSS (calcium, magnesium, no phenol red, 14025092, Gibco). Freshly isolated mitochondria from beetle larvae’s fat body (EX2620-100T, Solarbio) were incubated with 1 mL MSR under dark conditions at 37°C for 30 min. After triple washing with HBSS buffer, nuclear counterstaining was performed using DAPI (4',6-diamidino-2-phenylindole, D21490, Thermo Fisher Scientific) for 10 min at room temperature. The images were acquired using the Leica STELLARIS 5 confocal laser scanning microscopes. MSR reagent absorbs and emits optimally at 396 nm and 610 nm. DAPI absorbs and emits optimally at 348 nm and 454 nm.

*RNA Extraction, RNA Sequencing, Transcriptome and Phylogenetic Analysis:* A total of 6 libraries of *M. alternatus* were constructed from the subcutaneous fat body of recovered larvae (collected from February and reared under 25℃ for 4 weeks) injected with 5µL of 900nM (0.0045nmol) asc-C9 (recovery+C9) or vehicle (recovery+saline). A total of 12 libraries of mice were constructed from the BAT of mice injected with either vehicle (BAT-saline) or asc-C9 (BAT-C9), iWAT of mice injected with vehicle (iWAT-saline) or asc-C9 (iWAT-C9). Total RNA was isolated from the tissues using Trizol Reagent (Invitrogen, Carlsbad CA, USA) according to the manufacturer’s instructions. RNA sample concentration and purity were determined using a ND-1000 spectrophotometer (NanoDrop Technologies, Inc., Wilmington, USA). The RNA-seq library preparation kit for whole transcriptome discovery (-Illumina compatible) was used (Genome gene, San Diego, USA). The 320-420 bp products was purified using MiniElute gel extraction kit (Qiagen, Germany) and sequenced using the Illumina HiseqTM 2000 platform. All unigenes were annotated based on the BlastX results against non-redundant protein database (nr) at NCBI (http://www.ncbi.nlm.nih.gov/) and Swiss-prot database with the set e-value of < 10^-5^. The hierarchical clustering of all unigenes was performed with FPKM value (fragments kilobase of exon model per million mapped reads) more than 0.5. DEGs (differentially expressed genes) were identified by comparing libraries between uninfected to infected beetles, and transcripts with greater than 2-fold change (p < 0.001) were considered differentially expressed. Heat map analysis was performed by R package of pheatmap method (http://www.r-project.org/).

**Supplementary Figures**


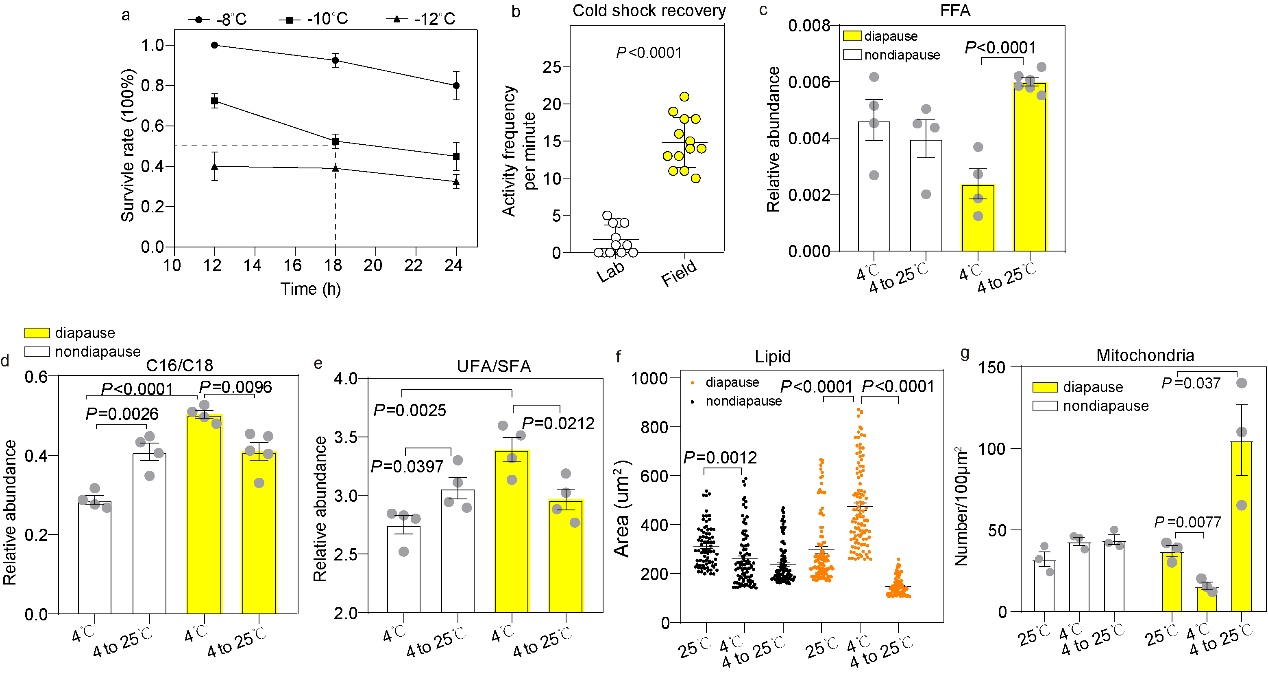


**Supplementary Figure 1.** The free fatty acid and acyl-CoA in diapause larvae and non-diapause larvae under cold stress of 4°C for12 week and recovery at 25°C for 1 week. a) The survival rate of pre-diapause larvae after 12h, 18h, 24h exposure to -8°C, -10°C, and -12°C. The lethal temperature required for 50% mortality of pre-diapause larvae (LTemp_50_) is marked with dotted line (with 95% Cl, *P*<0.05%). b) Vitality of field and lab larvae following exposure to -10 °C for 18 h and subsequent transfer to 25 °C for 24 h (n = 4 with 20 beetles/group). c-e) The free fatty acid level, C16/C18 ratio, and unsaturated/saturated ratio in non-diapause and diapause-larvae under cold stress of 4°C for12 week, and after transition from 4°C to 25°C for 1 week (n=4). f) The quantification of lipid area of HE staining in non-diapause and diapause-larvae under cold stress of 4°C for12 week, and after transition from 4°C to 25°C for 1 week (n=4). g) Quantification of mitochondrial number from transmission electron microscopy images in non-diapause and diapause larvae under the following conditions: maintained at 25°C, exposed to 4°C for 12 weeks, and returned to 25°C for 1 week following cold acclimation at 4°C (n = 3). Data are presented as mean±SD. *P* values were assessed by Student’s *t* test.


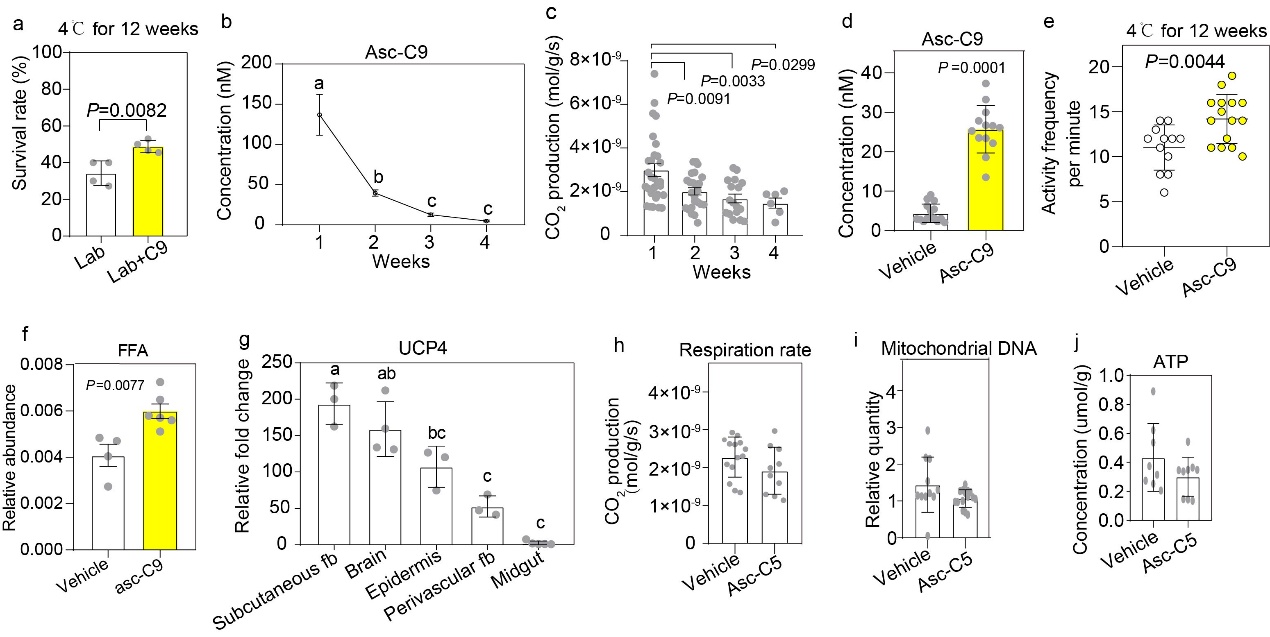


**Supplementary Figure 2.** Asc-C9 improves lipid mobilization in diapause larvae during recovering.

a) Survival rates of lab population injected with either vehicle or asc-C9 under 4°C for 12 weeks, followed by a recovery period at 25°C for one week (n=30). b) Asc-C9 concentration in recovering diapause larvae under 25^°^C treatment (n=20). c) The whole-body respiration rate of recovering diapause larvae under 25^°^C treatment (n=20). d) Asc-C9 concentration in recovered larvae before and after asc-C9 injection (n=20). e) Vitality of recovered larvae injected with either vehicle or asc-C9 under 4°C for 12 weeks, followed by a recovery period at 25°C for one week (n=30). f) The free fatty acid level of recovered larvae injected with either vehicle or asc-C9 (n=15). g) UCP4 expression in brain, subcutaneous fat body, epidermis, midgut and perivascular fat body of diapause larvae (n=5). h-j) The whole-body respiration rate, mtDNA copy number, and ATP concentration in recovered larvae injected with either vehicle or asc-C5 (n=3 with 5 beetles per group). Data are presented as mean±SD. *P* values were assessed by Student’s *t* test.


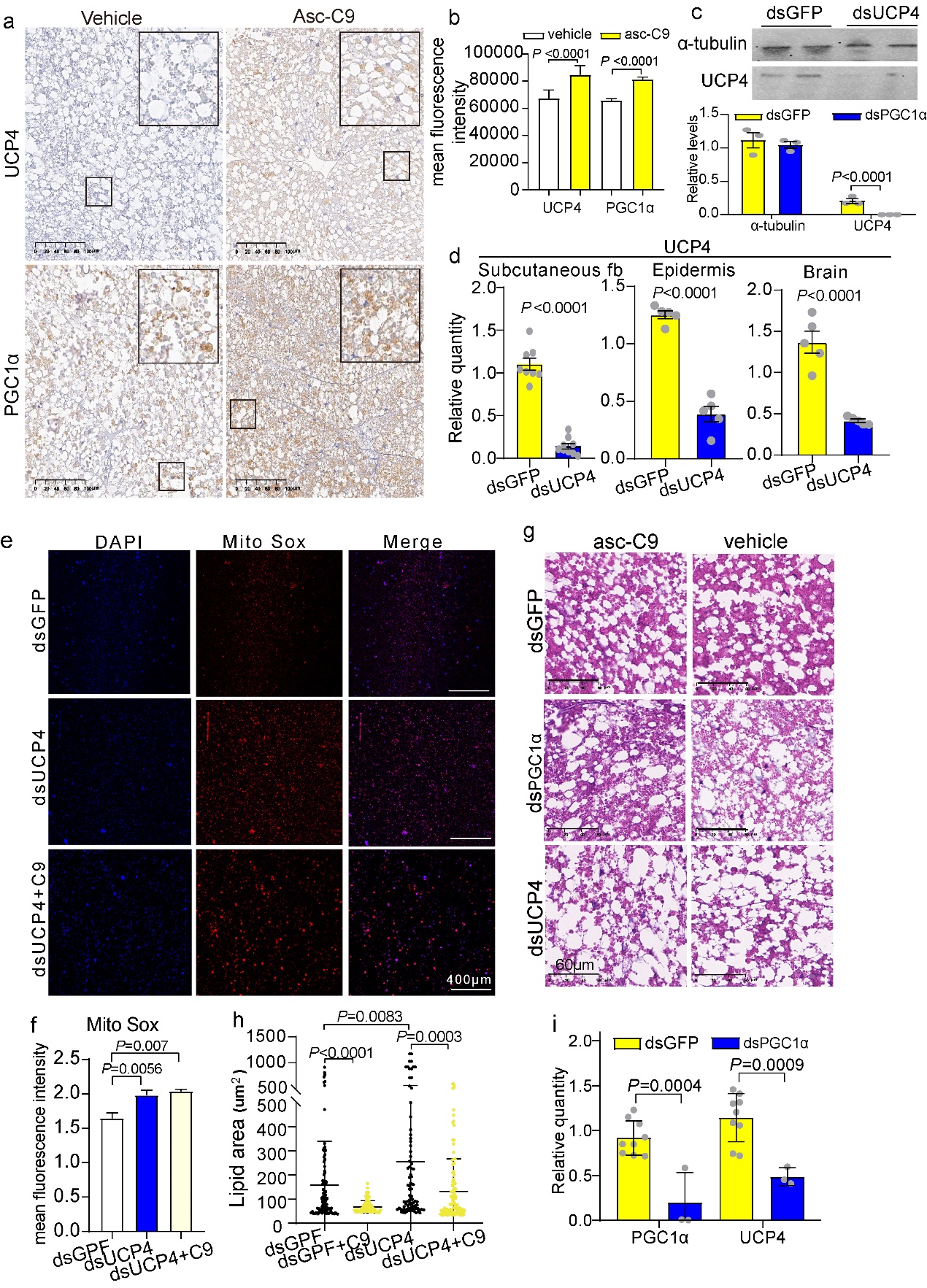


**Supplementary Figure 3.** Asc-C9 functions through UCP4 and PGC1α. a-b) The immunohistochemistry staining and mean fluorescence intensity analysis of UCP4 and PGC1α in sections of recovered larvae injected with either vehicle or asc-C9 (n=3). c) Western blot and quantification of UCP4 expression in subcutaneous fat body of GFP- and UCP4-depleted diapause larvae after transition from 4℃ to 25℃ (n=3). d) The relative mRNA quantity of UCP4 in subcutaneous fat body, epidermis, and brain of UCP4-depleted diapause larvae (n=10). e-f) The ROS level in mitochondria of GFP-, UCP4-, UCP4-depleted diapause larvae, and UCP4-depleted diapause larvae injected with asc-C9 after transition from 4℃ to 25℃ (n=3). g-h) The hematoxylin-eosin staining of subcutaneous fat body in GFP-depleted diapause larvae, and UCP4-depleted diapause larvae injected with either vehicle or asc-C9 after transition from 4℃ to 25℃ (n=3). i) The expression of UCP4 and PGC1α in GFP- and PGC1α-depleted diapause larvae after transition from 4℃ to 25℃ (n=10). Data are presented as mean±SD. *P* values were assessed by Student’s *t* test.


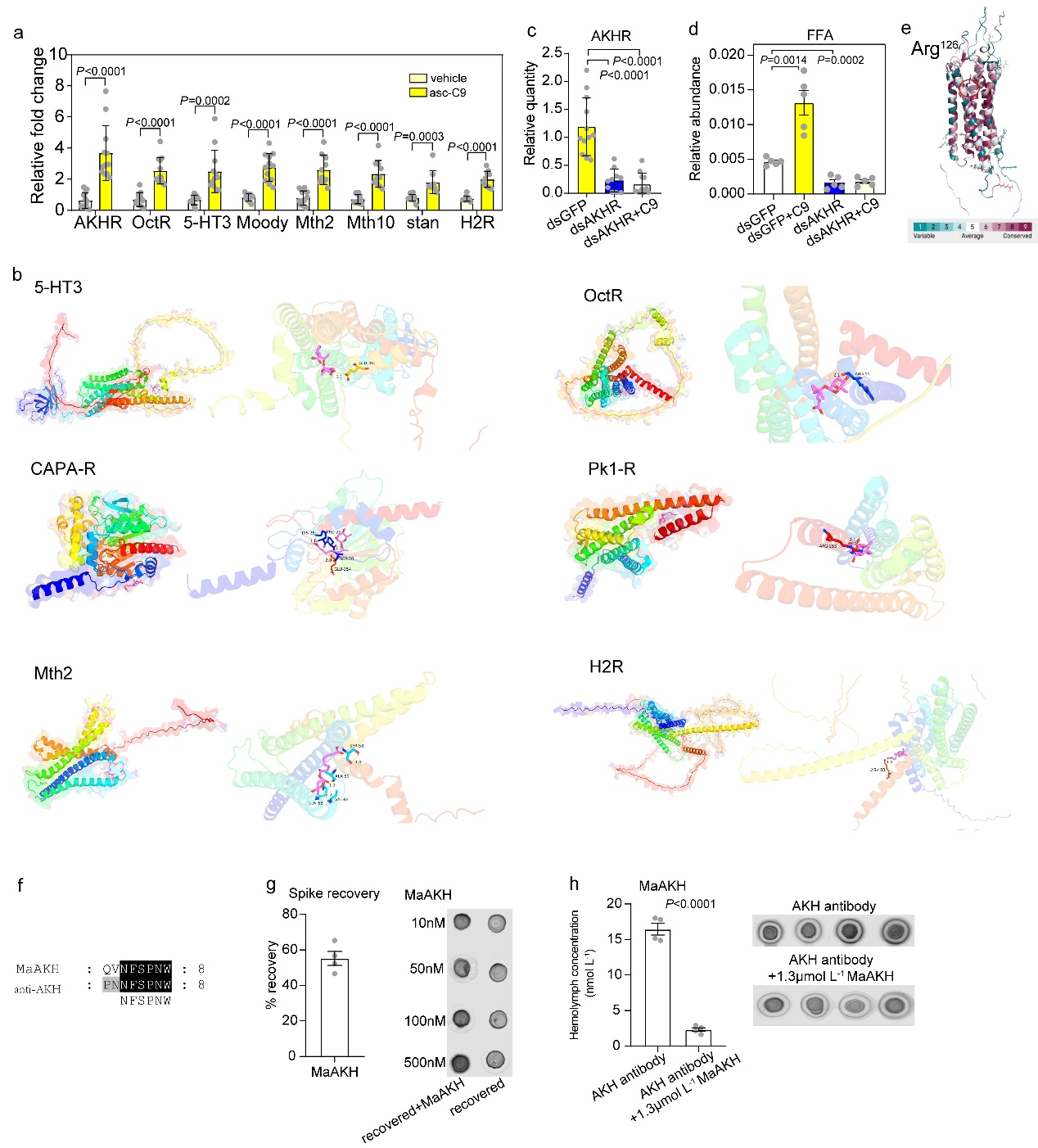


**Supplementary Figure 4.** Asc-C9 functions through AKHR. a) The qPCR analysis of receptor expression in recovery-type larvae injected with vehicle or asc-C9 (n=3 with 10 beetles per group). b) Binding poses of asc-C9 docked into GPCR candidate (OctR, 5-HT3, Pk1-R, CAPA-R, H2R, and Mth2) models. c) The AKHR expression in dsGFP-depleted, AKHR-depleted, and AKHR-depleted diapause larvae injected with asc-C9 (n=5 with 10 beetles per group). d) The free fatty acid level of dsGFP-, and AKHR-depleted diapause larvae injected with vehicle or asc-C9 (n=5 with 10 beetles per group). e) ConSurf Server-generated conservation analysis of the AKHR protein tertiary structure. The conserved residues of Arg^126^ are labeled. Data are presented as mean±SD. *P* values were assessed by Student’s *t* test. f) Sequence alignment of MaAKH and the antibody AKH. g) Spike-recovery assays of exogenous MaAKH added to hemolymph samples. h) Dot-blot analysis of asc-C9 injected hemolymph samples using AKH antibody alone (control) or AKH antibody pre-incubated with 1.3 µM synthetic MaAKH.


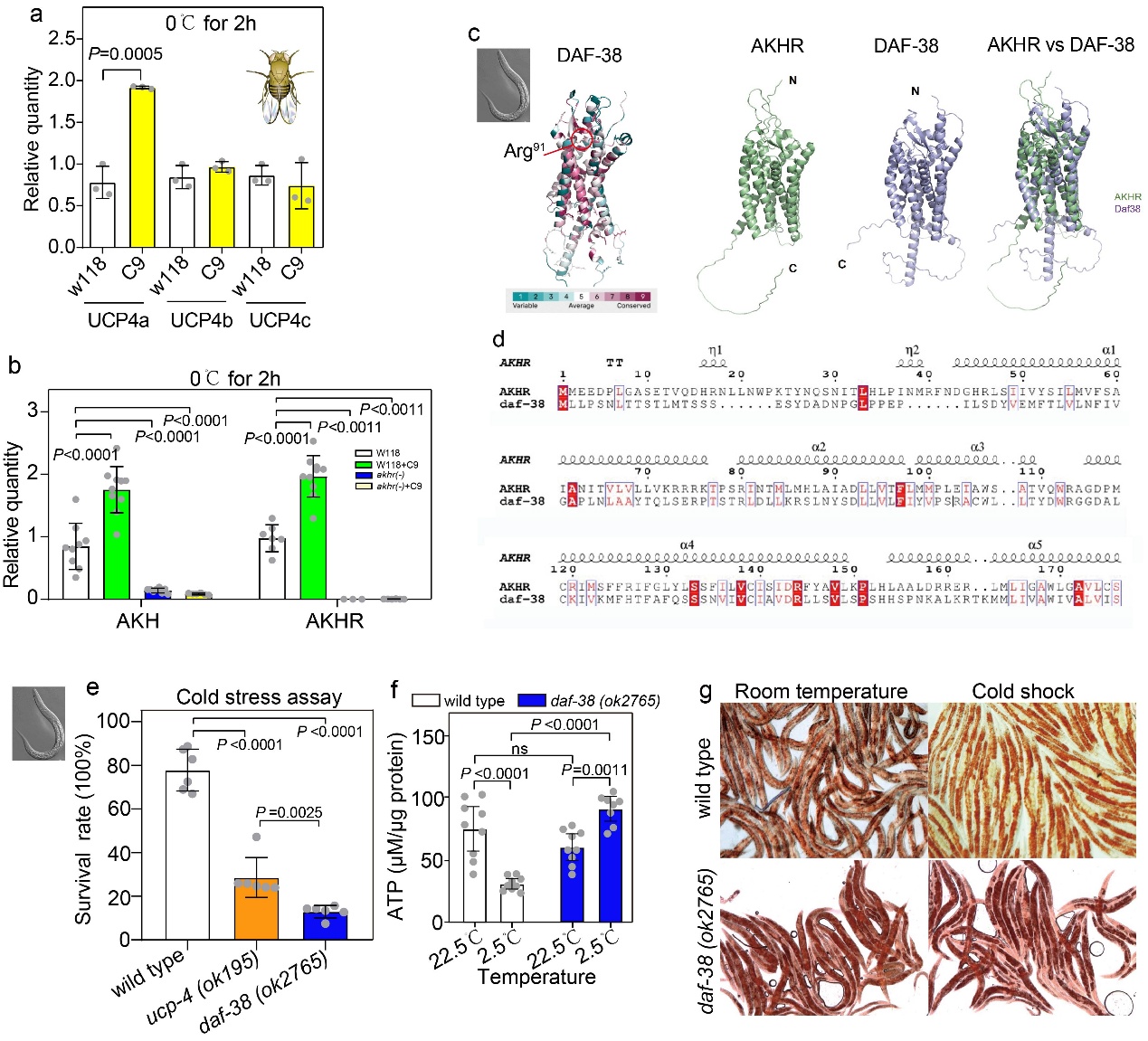


**Supplementary Figure 5.** Asc-C9 functions through AKHR. a) The expression of UCP4a, UCP4b, and UCP4c after cold shock (at 0^°^C for 2h) of wild-type *D. melanogaster* fed with either vehicle or asc-C9 (n=3). b) Gene expression of AKH and AKHR in wild-type, *akhr-*mutant *D. melanogaster* fed with either vehicle or asc-C9 under cold shock at 0^°^C for 2h (n=8-10). c) ConSurf Server-generated conservation analysis of the DAF-38 protein tertiary structure, with conserved residue Arg^91^ labeled. Similarity analysis of the predicted structures of AKHR and DAF-38 using AlphaFold. d) Comparison of amino acid sequences and tertiary structures between DAF-18 and AKHR. e) The survival of wild type, *ucp-4(ok195)*, and *daf-38(ok2765)* *Caenorhabditis elegans* in cold stress assay (n=3 with three per group). f) The ATP levels of wild type, *ucp-4(ok195)*, and *daf-38(ok2765)* *C. elegans* at room temperature (22.5 °C) and low temperature (2.5 °C) (n = 9 with three per group). g) The fat levels of wild type and *daf-38(ok2765)* *C. elegans* at room temperature (22.5 °C) and low temperature (2.5 °C) (n = 3 with three per group). Data are presented as mean±SD. *P* values were assessed by Student’s *t* test.

Reference

[1] W. B. Iser, D. Kim, E. Bachman, C. Wolkow, *Mechanisms of ageing and development* **2005**, *126* (10), 1090, <https://doi.org/10.1016/j.mad.2005.04.002>.
